# Nanotechnology for next-generation CAR T-Cell manufacturing

**DOI:** 10.3389/fimmu.2026.1896088

**Published:** 2026-10-21

**Authors:** Mark Schvartzman, Saba Ghassemi

**Affiliations:** 1 Department of Materials Engineering, Ben-Gurion University of the Negev, Beer-Sheva, Israel; 2 Ilse Katz Institute for the Nanoscale Science and Technology, Faculty of Engineering, Ben-Gurion University of the Negev, Beer-Sheva, Israel; 3 Center for Cellular Immunotherapies, Perelman School of Medicine, University of Pennsylvania, Philadelphia, PA, United States; 4 Department of Pathology and Laboratory Medicine, Perelman School of Medicine, University of Pennsylvania, Philadelphia, PA, United States

**Keywords:** cancer, CAR T cells, immunotherapy, nanomaterials, nanotechnology

## Abstract

Chimeric antigen receptor (CAR) T-cell therapy has transformed the treatment landscape of hematological malignancies; however, its broader clinical implementation remains constrained by the complexity, cost, and variability of current manufacturing workflows. Conventional CAR T-cell production relies on multistep ex vivo processes including T-cell activation, genetic engineering, expansion, and quality control, each of which influences the phenotype, persistence, and therapeutic efficacy of the final cellular product. Increasing evidence indicates that manufacturing is not merely a logistical process, but a critical biological determinant of CAR T-cell function. Consequently, the field is undergoing a conceptual transition from maximizing expansion and transduction toward actively engineering therapeutically favorable cellular states. Recent advances in nanotechnology and biomaterials engineering provide unprecedented opportunities to address these challenges through precise control of biochemical, spatial, and mechanical cues governing T-cell behavior. This review discusses emerging nanotechnology-enabled strategies across the complete CAR T-cell manufacturing pipeline. The review examines engineered activation platforms that exploit substrate stiffness, topography, ligand organization, and mechanobiological signaling to tune T-cell activation, differentiation, and exhaustion. It further discusses nanotechnology-based approaches for improving viral transduction and enabling non-viral CAR delivery through lipid nanoparticles, nanostructured interfaces, and intracellular delivery systems. Emerging strategies for engineering expansion microenvironments designed to preserve stem-like and memory phenotypes, as well as *in vivo* CAR generation strategies that may bypass conventional ex vivo manufacturing altogether, are then highlighted. Finally, we highlight nanoscale biosensing and analytical platforms that enable real-time manufacturing monitoring, quality control and adaptive process optimization. Collectively, these advances position nanotechnology as a transformative platform for next-generation CAR T-cell manufacturing.

## 1 Introduction

Chimeric antigen receptor (CAR) T-cell therapy has emerged as a transformative approach in cancer immunotherapy, producing unprecedented clinical responses in patients with otherwise refractory hematologic malignancies (1). CARs are synthetic receptors that redirect T cells toward tumor-associated antigens in a major histocompatibility complex (MHC)-independent manner, enabling potent and selective tumor killing. The first CAR design consisted of an extracellular antigen-recognition domain, most commonly derived from a single-chain variable fragment (scFv), linked through a hinge and transmembrane region to intracellular signaling domains responsible for T-cell activation and stimulation (2). The incorporation of costimulatory domains such as CD28 or 4-1BB has substantially improved CAR T-cell expansion, persistence, metabolism, and long-term anti-tumor activity (3). These advances have led to multiple FDA-approved CAR T-cell therapies for B-cell malignancies and multiple myeloma, establishing CAR T cells as a new therapeutic pillar in oncology (4).

Today, CARs are commonly classified into five generations according to the intracellular signaling modules incorporated into the receptor (5, 6). First-generation CARs contain CD3ζ alone, second-generation CARs combine CD3ζ with a single costimulatory domain such as CD28 or 4-1BB, and third-generation CARs incorporate two costimulatory domains. Fourth- and fifth-generation CARs extend this framework functionally, although they are not necessarily simple cumulative descendants of third-generation receptors (**Figure 1a**). Rather, fourth-generation CARs are typically based on a second-generation backbone coupled to an inducible payload, such as cytokine secretion, to modulate the tumor microenvironment, whereas fifth-generation CARs generally add a cytokine-receptor signaling module, commonly based on IL-2Rβ/STAT3/5 recruitment, to provide antigen-triggered JAK-STAT signaling.

**Figure 1:**
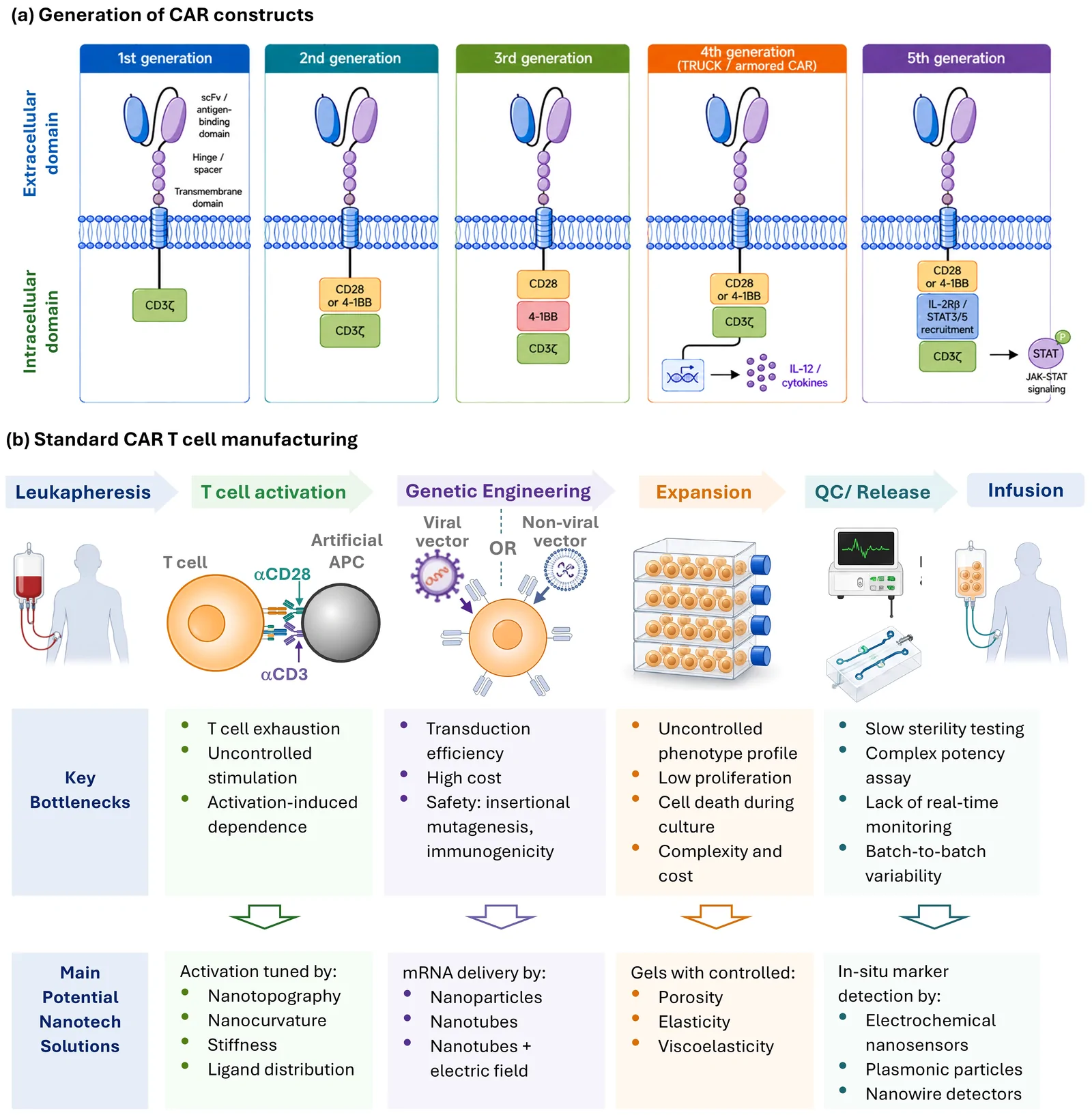
**(a)** Five generations of CARs **(b)** workflow of the state-of-the-art ex-vivo CAR T-cell manufacturing, including key bottle necks and challenges of each step, and potential nanomaterial based solutions.

Clinically, CAR T-cell therapy has achieved its greatest impact in hematological malignancies. As of 2026, seven CAR T-cell products have received FDA approval, including five targeting CD19 and two targeting B-cell maturation antigen (BCMA), with indications spanning B-cell acute lymphoblastic leukemia, several forms of B-cell lymphoma, chronic lymphocytic leukemia, and multiple myeloma (7). These therapies have produced high response rates in heavily pretreated and relapsed/refractory patients, with CD19-directed CAR T cells inducing prolonged remissions that can persist for many years and appear to be curative in a subset of patients, while BCMA-directed CAR T cells have established an effective treatment modality for relapsed or refractory multiple myeloma, although response durability is generally more limited (8). Thus, CAR T-cell therapy has progressed from an experimental form of adoptive cell therapy to an established therapeutic platform across multiple B-cell malignancies, although its clinical efficacy in solid tumors remains substantially less developed. In parallel, advances in mRNA-based cancer immunotherapy, including stimuli-responsive nanomaterials for mRNA vaccine delivery and AI-assisted optimization of tumor-antigen selection and vaccine design, illustrate the broader convergence of nanotechnology, nucleic-acid therapeutics, and programmable immune modulation in next-generation cancer immunotherapy (9, 10).

Despite these remarkable successes, extending the efficacy of CAR T-cell therapy to broader patient populations and solid tumors remains a major challenge. Tumor heterogeneity, antigen escape, immunosuppressive tumor microenvironments, and limited CAR T-cell persistence continue to restrict durable responses. Increasing evidence suggests that therapeutic efficacy is not solely determined by CAR design itself, but also by the biological state of the manufactured T cells prior to infusion (11). Unlike conventional pharmaceuticals, CAR T cells are living drugs whose phenotype, metabolic fitness, differentiation state, and functional durability are heavily influenced by the ex vivo manufacturing process (12). As a result, CAR T-cell manufacturing has evolved from a purely logistical process into a critical determinant of therapeutic potency and clinical outcome.

Although manufacturing workflows vary across clinical products, most currently approved CAR T-cell therapies rely on a broadly similar ex vivo manufacturing paradigm involving leukapheresis, T-cell activation, genetic engineering, expansion, and cryopreservation prior to reinfusion (**Figure 1b**). Each of these steps introduces distinct biological and engineering challenges that can significantly influence the quality, consistency, and efficacy of the final cellular product. In addition to being labor-intensive and highly individualized, current manufacturing approaches are associated with prolonged vein-to-vein times, substantial costs, manufacturing failures, and limited scalability, all of which restrict patient accessibility and present major barriers for rapidly progressing disease.

The manufacturing process typically begins with leukapheresis, during which peripheral blood mononuclear cells are collected from the patient. However, heavily pretreated patients frequently exhibit poor T-cell quality, low lymphocyte counts, or features of immune dysfunction and exhaustion, creating variability in the starting material even before manufacturing begins (13). Isolated T cells are then activated ex vivo, most commonly using anti-CD3/CD28 antibody-coated beads or artificial antigen-presenting systems designed to mimic physiologic T-cell stimulation. Activation is required to induce metabolic reprogramming, proliferation, and increased permissiveness to gene transfer. However, strong T-cell receptor (TCR)-mediated activation also initiates differentiation programs that progressively drive T cells away from naïve and stem-like states toward more terminally differentiated phenotypes (14–16). While these differentiated cells exhibit potent short-term cytotoxicity, they often demonstrate impaired persistence, reduced proliferative capacity, and increased susceptibility to exhaustion following infusion (17, 18).

Following activation, T cells are genetically engineered to express the CAR transgene, most commonly using lentiviral or gamma-retroviral vectors that enable stable genomic integration and long-term CAR expression. Viral gene delivery remains the clinical standard due to its relatively high efficiency and durable transgene expression; however, it is associated with substantial manufacturing complexity, high production costs, regulatory burden, and variability between donors and production runs. Alternative non-viral approaches, including electroporation-based mRNA delivery and transposon systems, have emerged as promising strategies to simplify manufacturing and improve safety, although many remain limited by transient expression, delivery efficiency, or scalability challenges. Importantly, the efficiency and mode of gene delivery can themselves influence T-cell activation state, viability, and downstream therapeutic function (19–22).

The final major stage of conventional CAR T-cell manufacturing involves ex vivo expansion, during which engineered cells are cultured for days to weeks to achieve clinically relevant doses for infusion. Although conventional manufacturing workflows incorporate ex vivo expansion to increase final cell yield, prolonged culture is increasingly recognized as a major driver of T-cell differentiation, metabolic dysfunction, and exhaustion. Extended ex vivo proliferation promotes metabolic stress, differentiation, loss of stem-like and central memory subsets, and acquisition of exhaustion-associated phenotypes that may compromise long-term anti-tumor efficacy (23–25). In addition, the expansion phase substantially contributes to manufacturing time, cost, and logistical complexity. Most current workflows also incorporate cryopreservation steps prior to infusion, which can further impact cell viability, recovery, and functional fitness following thawing. Collectively, these limitations have fueled growing interest in strategies aimed at shortening manufacturing duration, preserving less differentiated T-cell states, and improving overall product consistency and potency.

Importantly, the autologous nature of current CAR T-cell therapies provides a key safety advantage by minimizing the risks of immune rejection and graft-versus-host disease. However, the patient-specific nature of these therapies limits scalability and manufacturing throughput and contributes to the high cost and complexity of production (26). Emerging allogeneic and *in vivo* engineering strategies may overcome some of these limitations but introduce distinct safety considerations that must be carefully addressed during clinical translation (27).

Recent advances in nanotechnology and nanomaterial engineering offer new opportunities to address many of these manufacturing bottlenecks. While the original boost in nanotechnology over the last three decades was primarily motivated by the need to address emerging gaps in the field of solid-state electronics, the newly developed tools enabling control and structuring of matter at the nanometric scale have rapidly entered the realm of biological applications. This is because these tools can precisely control biological objects that span the nanometric size range—from individual biomolecules sized from a few to a few tens of nanometers, up to abundant biological objects sized from hundreds of nanometers to one micron, such as viruses, organelles, small bacteria, and cell filopodia. This level of control, which is unachievable by conventional methods in experimental cell biology and biomedicine, enables unprecedented regulation of fundamental cellular functions, including but not limited to adhesion, motility, proliferation, differentiation, and cell death.

While these tools have been broadly applied in biology and biomedicine over the last two decades, they have not bypassed T-cell engineering, and in particular, CAR T-cell engineering. Nanotechnology-based platforms can provide precise control over the biochemical, mechanical, and spatial cues that regulate T-cell activation, gene delivery, expansion, and differentiation.

Engineered nanoscale materials have been explored to improve viral and non-viral transduction efficiency, create biomimetic activation environments, modulate T-cell mechanobiology, enhance the expansion of therapeutically favorable subsets, and even enable *in vivo* CAR T-cell generation. These approaches aim not only to improve manufacturing efficiency, but also to actively program CAR T-cell function and therapeutic quality.

It should be noted that applications of nanomaterials in CAR T-cell immunotherapy are emerging and highly diverse. These include, in particular, nanomaterials for overcoming the immunosuppressive tumor microenvironment (28, 29); surface engineering of CAR T cells with nanomaterials that act as local carriers for checkpoint inhibitors, chemotherapeutic drugs, and imaging agents (30–33); nanomaterials for improved tumor homing, e.g., through magnetic guidance or localized inflammatory stimulation (34, 35); and nanomaterials for CAR T-cell monitoring and tracking, e.g., fluorescent probes or magnetic nanoparticles for MRI tracking (33, 36, 37). These and related applications have been extensively discussed in recent reviews of nanotechnology and nanomaterials for T-cell and CAR T-cell immunotherapy (38–41). While some of these reviews also address aspects of CAR T-cell production, their broader emphasis is generally placed on the use of nanomaterials throughout the therapeutic process, including enhancement of T-cell function *in vivo*, tumor targeting and infiltration, modulation of the tumor microenvironment, and direct *in vivo* immune-cell engineering. In contrast, the present review adopts CAR T-cell manufacturing itself as its central organizing framework. Rather than considering nanotechnology primarily as an adjunct to the therapeutic activity of already engineered T cells, here we examine how nanoscale materials and engineering principles can intervene at each major stage of CAR T-cell production—from activation and genetic engineering to expansion, *in vivo* generation, and manufacturing monitoring and quality control. A particular emphasis is placed on the ability of engineered materials to control not only conventional manufacturing metrics such as cell yield and transduction efficiency, but also the biological quality of the resulting cellular product, including activation strength, differentiation state, exhaustion, memory phenotype, metabolic fitness, persistence, and ultimately therapeutic potency (**Box 1**). Accordingly, nanoscale biochemical organization, topography, stiffness, viscoelasticity, and other physical cues are considered here as programmable manufacturing parameters rather than simply material properties. This manufacturing-centered perspective therefore positions nanotechnology as a platform for transitioning CAR T-cell production from a largely empirical process aimed at generating sufficient quantities of engineered cells toward a rationally engineered process that simultaneously optimizes cell state, product consistency, scalability, accessibility, and therapeutic efficacy.

Box 1 **From maximal expansion to functional optimization - conceptual shift in CAR T-cell manufacturing**

Historically, CAR T-cell manufacturing has largely prioritized maximal expansion, high transduction efficiency, and standardized production workflows. Conventional manufacturing paradigms were developed to reliably generate large numbers of engineered T cells suitable for clinical infusion, often relying on strong ex vivo activation, prolonged culture, and extensive cellular manipulation. While these approaches enabled the clinical translation and commercialization of CAR T-cell therapies, growing evidence suggests that intensive ex vivo processing can profoundly alter T-cell biology in ways that may compromise long-term therapeutic efficacy.

Prolonged activation and expansion drive progressive T-cell differentiation, metabolic rewiring, and acquisition of exhaustion-associated phenotypes, often at the expense of naïve, stem-like, and central memory populations that are associated with durable persistence and sustained anti-tumor responses. Increasing recognition that CAR T-cell therapeutic performance is tightly linked to cellular state has shifted attention toward manufacturing strategies that aim not only to maximize cell yield, but also to preserve or actively program favorable T-cell phenotypes during production.

This evolving perspective has led to growing interest in manufacturing paradigms centered on minimizing ex vivo stress while maintaining efficient gene transfer and therapeutic potency. Emerging approaches include shortened manufacturing workflows, reduced or activation-free engineering strategies, biomimetic activation systems, and engineered microenvironments designed to more closely replicate physiological T-cell priming conditions. Rather than viewing manufacturing solely as a logistical process, these strategies increasingly treat manufacturing as a programmable biological engineering platform capable of shaping T-cell fate, metabolism, differentiation, and function.

Nanotechnology and biomaterials engineering are particularly well positioned to enable this transition. By providing precise control over nanoscale biochemical, mechanical, and spatial cues, engineered materials can modulate T-cell activation strength, receptor organization, mechanotransduction, gene delivery, and expansion dynamics with a level of precision that is difficult to achieve using conventional manufacturing approaches. In this emerging framework, nanotechnology is not simply a supportive manufacturing tool, but a central platform for engineering next-generation CAR T-cell therapies with improved potency, persistence, scalability, and accessibility.

Each section of this review corresponds to a specific step of CAR T-cell manufacturing. The first section is dedicated to CAR T-cell activation. It first discusses the fundamentals of T-cell activation and compares physiological T-cell activation by target cells or antigen-presenting cells with activation by artificial activating platforms. It further outlines the effects of the physical features of the activating microenvironment—mechanical properties, morphology, and spatial distribution of activating ligands—on T-cell response. This is followed by a review of recent studies in which these features are harnessed to engineer controllable T-cell activation platforms for optimized CAR T-cell manufacturing. The next section is dedicated to the genetic engineering of T cells and focuses on nanotechnology-based approaches to improve both viral transduction and non-viral transfection for CAR expression. In each of these sections, nanomaterial based solutions explicitly designed to facilitate each specific step in CAR T cells manufacturing are discussed (**Table 1**). The following section describes emerging strategies for engineering the CAR T-cell expansion microenvironment, aimed not only at increasing the yield of produced CAR T cells, but also at steering their phenotypic composition, which strongly influences the immunotherapeutic outcome after infusion. Finally, the last section describes emerging nanomaterial-based strategies for the *in vivo* generation of CAR T cells, which is currently viewed as a potential strategy to reduce manufacturing time, cost, and complexity relative to conventional ex vivo approaches. Overall, this review highlights the emerging role of nanomaterials and nanotechnology not only as enabling tools for improving individual steps of CAR T-cell manufacturing, but also as transformative platforms with the potential to redefine the scalability, accessibility, and therapeutic performance of next-generation CAR T-cell immunotherapies.

**Table 1:** Nanomaterial bases solutions demonstrated to improve CAR T cell manufacturing.

Production step	Nanomaterial/nanotechnology	Major advance/achievement	Ref.
Activation	T Cell TransAct™ colloidal nanomatrix (~100 nm iron-oxide/polysaccharide particles carrying anti-CD3/anti-CD28)	Bead-removal-free activation; compatible with closed/automated manufacturing	(42)
	Lipid-coated mesoporous silica nanorod scaffolds carrying anti-CD3/CD28 and releasable paracrine factors	Strong CAR T restimulation and controllable stimulation dose	(43, 44)
	Ligand-functionalized PDMS nanopillar surfaces with optimized stiffness/topography	Improved activation/transduction, reduced exhaustion, increased central memory and antitumor efficacy	(45, 46)
	Viscoelastic synthetic APC particles with tunable stress relaxation	TSCM enrichment; ~90% CAR transduction; improved persistence/tumor control	(47)
Transduction/Transfection	Polycaprolactone nanoporous substrate for lentiviral transduction	~500-nm pores enhance viral uptake and CAR T cytotoxicity	(48)
	Glycometabolic bioorthogonal virus targeting — azide-labelled T cells + DBCO-functionalized lentivirus	Targeted viral binding; improved CAR T antitumor efficacy	(49)
	Ionizable lipid nanoparticles (LNPs) for CAR-mRNA delivery	CAR expression comparable to electroporation with lower cytotoxicity	(50)
	Composition-optimized ionizable LNPs	Improved CAR-mRNA delivery efficiency	(51)
	Multicargo ionizable LNPs carrying CAR mRNA + PD-1 siRNA	Simultaneous CAR expression and transient PD-1 knockdown	(51)
	APC-mimetic LNPs carrying CAR mRNA and surface anti-CD3/costimulatory antibodies	Combines activation + CAR delivery; eliminates separate bead activation	(52)
	Silicon nanotube arrays loaded with CAR expression cassettes	Passive, non-viral CAR delivery; functional CAR T cells	(53)
	Electrically assisted silicon nanotube/nanostraw arrays	~70% transfection with >90% viability	(54)
Proliferation/expansion	Microporous PEG–heparin hydrogel with tunable pore size/stiffness	~2-fold higher CAR T replication index	(55)
	Self-assembling peptide gel with optimized stiffness	~10-fold greater expansion and shorter proliferation time	(56)
	Viscoelastic collagen-based 3D niche with tunable stress relaxation	Mechanical control of memory-like vs effector CAR T phenotype	(57)
	Soft 3D fibrin gel niche	Enriches stem-like CAR T cells; improved tumor control	(15)

## 2 Nanotechnology for the ex-vivo T-Cell activation

### 2.1 Ex-vivo T-Cell activation – state of the art

Ex vivo activation of T cells is a gate-opening step in CAR T-cell manufacturing. It renders T cells permissive to genetic modification, initiates robust proliferation to clinically relevant cell numbers, and establishes the functional state of the final therapeutic product. Importantly, the strength, duration, and context of the activation signals profoundly influence T-cell differentiation, exhaustion, metabolic fitness, and long-term persistence *in vivo*. Thus, T-cell activation is not merely a preparatory step in CAR T-cell production, but a key determinant of CAR T-cell quality, efficacy, and durability following infusion. Interestingly, CAR T-cell manufacturing lacking this “pre-requisite” activation step is an effective approach to shorten vein-to-vein time and achieve higher antitumor persistence; however, the tradeoff is low transduction efficiency (58). Therefore, activation is still broadly considered an essential step in CAR T-cell manufacturing.

All existing activation platforms are, in principle, designed to mimic *in vivo* T-cell activation, specifically its key elements—triggering the TCR/CD3 complex (signal 1) and engaging costimulatory CD28 receptors (signal 2) (**Figure 2**). While *in vivo* these receptors are triggered by binding to their cognate ligands expressed by antigen-presenting cells (APCs) or target cells, their complementary ex vivo counterparts rely on antibodies against these receptors. These antibodies are immobilized to a solid support such as a magnetic bead, tetrameric complex or artificial APC. While both antibody- and endogenous ligand-based triggering activate the same signaling pathways, resulting in similar functional outcomes, they differ substantially in their kinetics. The relatively low pMHC–TCR affinity results in a high on–off binding rate, enabling a serial triggering mechanism by which one ligand can activate multiple receptors, and as little as one ligand can be sufficient to activate an entire T cell (59, 60). This mechanism is not possible in antibody-based activation, whose affinity to the receptor is several orders of magnitude higher. Therefore, the number of antibodies required to activate a T cell is much higher than that of ligands (61).

**Figure 2:**
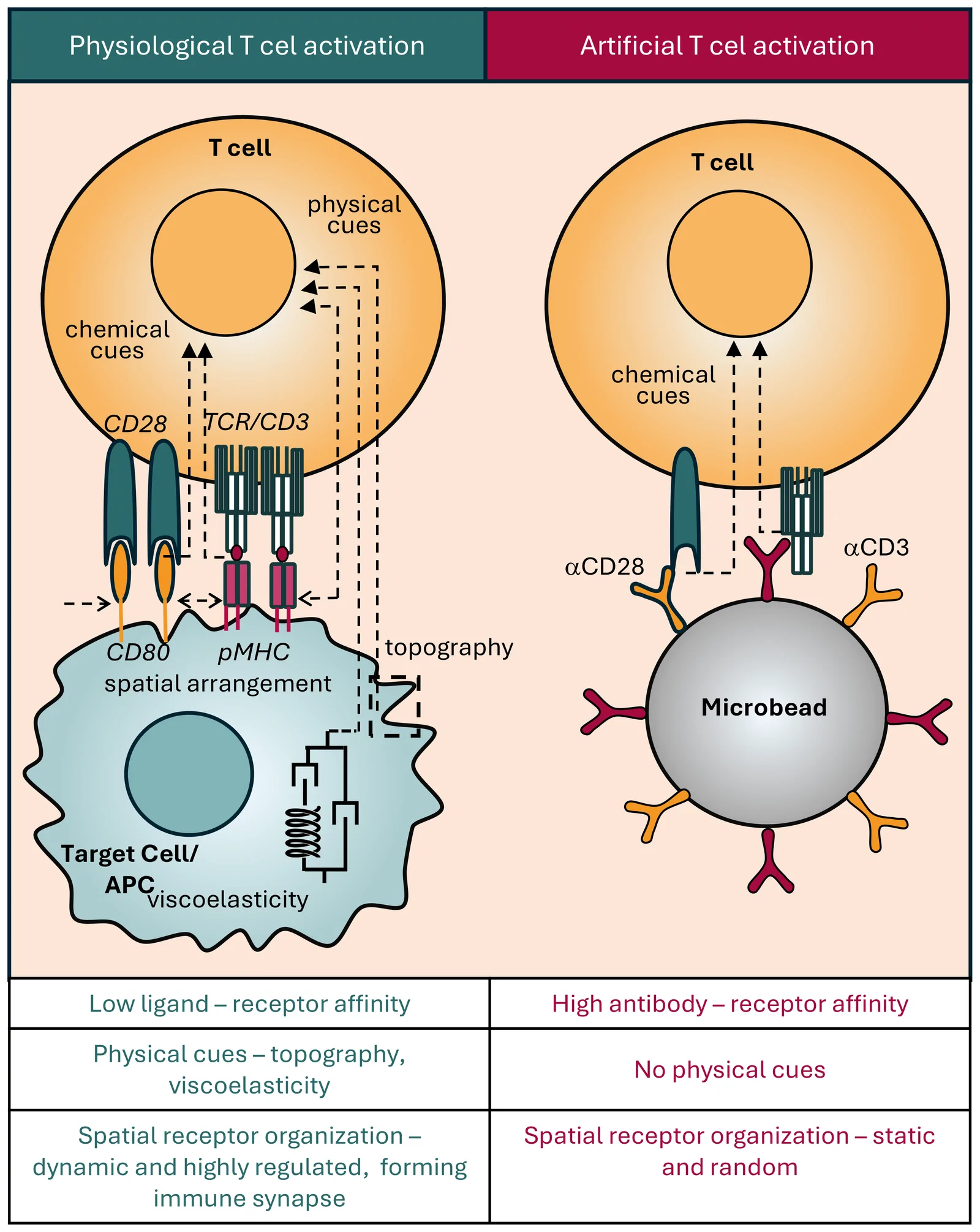
Comparison between physiological T-cell activation and T-cell activation by an artificial signal carrying platform, emphasizing the main differences in presenting physical cues.

Another essential feature of T-cell receptor triggering is that the activating molecules—ligands for physiological activation or antibodies for ex vivo activation—must be tethered to a solid support. Liquid antibodies can, in principle, activate T cells, but with limited efficiency (62). T cells can release from a quiescent state following CD3 engagement but fail to undergo cell division (63). The requirement for a solid support stems from the fundamental mechanism of T-cell activation via receptor binding and force generation. Notably, while the exact mechanism is still highly debated (64, 65), most proposed mechanisms agree that activating molecules must be immobilized on a solid support (66, 67).

In the CAR T-cell generation process, T cells are most commonly activated using artificial APCs in the form of beads functionalized with antibodies against CD3 and CD28. A prominent example of such a commercially available product is Dynabeads™, 4.5 micron-sized beads with a polystyrene shell and an iron oxide-based core, which enables magnetic separation from the produced CAR T cells, usually at the end of the proliferation stage. Interestingly, Dynabeads were developed in the late 1970s, originally to enable highly controlled magnetic separation of biomolecules, proteins, and cells, and only later were adopted for T-cell activation.

More recently, removable or biodegradable nanoscale activation matrices have emerged as alternatives to conventional micron-sized magnetic beads, eliminating the need for a dedicated magnetic bead-removal step. A prominent example is T Cell TransAct™ (Miltenyi Biotec), a colloidal nanomatrix of approximately 100 nm comprising nanoscale iron-oxide crystals embedded within a biocompatible polysaccharide matrix, to which agonistic anti-CD3 and anti-CD28 antibodies are covalently conjugated (42). Owing to its small size and dispersible structure, excess TransAct can be removed during routine medium exchange or washing/centrifugation, rather than by magnetic separation, facilitating its incorporation into closed and automated CAR T-cell manufacturing workflows. The polymeric matrix is frequently described as biodegradable; however, its precise polysaccharide composition and molecular degradation mechanism have not been publicly disclosed.

### 2.2 The role of the physical parameters of the activating environment

While these materials are commercially available and provide T cells with essential biochemical signals through immobilized antibodies, their design has not taken into consideration the physical stimuli of the *in vivo* T-cell activating environment. These characteristics have been shown to be critical in determining the outcome of T-cell activation. The main characteristics are:

- Elasticity. *In vivo*, the T-cell environment is characterized by high mechanical diversity. In recent years, it has become increasingly clear that T cells possess mechanosensing mechanisms, which they use to differentiate between their targets and healthy cells and tissues (68, 69). This hypothesis has been confirmed by several ex vivo studies showing that T-cell responses vary significantly with the elasticity of the ligand-supporting surface (70, 71).
- Topography. The T-cell–target interface has a complex topographic landscape (72, 73). T cell interact with their targets by microvilli -- nanoscale protrusions enriched with actin filaments, that form a quasi-contractile apparatus (74). The tips of the finger-like projections are enriched with TCR and proximal signaling molecules (75). The mechanism of microvilli sensing is under investigation, and their high localized curvature is hypothesized to facilitate segregation of CD45 phosphatase from the microvilli tips, thereby increasing T-cell sensitivity (74, 76). Interestingly, nanoscale topography of activating surfaces can induce membrane protrusions in T cells, resulting in a certain, though minor, level of T-cell activation (77, 78). The mechanism of this ligand-independent activation is unclear but is presumed to arise from TCR–CD45 segregation induced by membrane curvature (78).
- Ligand composition and spatial organization. Ligands are diffusely distributed across the target cell surface. Following contact with T cells and binding to TCRs, dynamic nanoclusters (~200–500 nm) form (79). This interface then matures into a bull’s-eye structure with radial symmetry, known as the immune synapse, consisting of: a central supramolecular activation cluster (cSMAC), into which TCR–ligand clusters translocate and integrate with costimulatory CD28 clusters for sustained signaling and eventual TCR downregulation/internalization; a surrounding peripheral SMAC (pSMAC), which hosts LFA-1/ICAM-1 adhesion rings that stabilize the contact and enable centripetal microcluster transport via actin/myosin IIA flow; and a distal SMAC (dSMAC), containing primarily the large tyrosine phosphatase CD45 and the adhesion molecule CD43 (80, 81). Ex vivo studies using ligands patterned into controlled arrays show that parameters such as surface density, separation, TCR internalization/recycling and even clustering independently “tonically” in the absence of stimuli regulate T-cell activation and can deterministically tune the T-cell response (82–86).

Naturally, an ideal T-cell activation platform can and should be designed while considering these physical stimuli. This approach will not only bridge ex vivo T-cell activation to mimic *in vivo* conditions but also allow tuning of T-cell responses and their functional outcomes, up to the level of *in-vivo* antitumor efficacy. Importantly, while most studies of material-based approaches have focused on driving high levels of activation, maximal activation is not necessarily the optimal goal. Overactivation induces hallmark exhaustion markers (PD-1, TIM-3, LAG-3, TOX), which in turn reduce cytokine production and diminish proliferative capacity (87).

In addition, T-cell differentiation strongly determines antitumor function. Specifically, the central memory phenotype is associated with enhanced antitumor functionality (15, 88, 89). Overall, a desired stimulation platform should achieve an optimal balance among the following factors: (i) activation level sufficient for high transduction efficiency and proliferation, (ii) low exhaustion, and (iii) tuned differentiation as well as metabolism toward phenotypes relevant for persistent antitumor efficacy. Based on recent research, the physical characteristics of the T-cell stimulating environment can be regarded as “tuning knobs” of the stimulation process to achieve this balance. The next sections review recent innovations in improving T-cell activation outcomes through the physical characteristics of the material on which the activating molecules are immobilized, with an emphasis on CAR T-cell generation.

### 2.3 Nanomaterial platforms for tuning mechanical stimulation of T cells

Materials with modulated stiffness have initially been used for T-cell activation to mimic the mechanical diversity of the natural T-cell environment and to study T-cell responses to this diversity, as well as how T cells use mechanosensing to discriminate between cancerous, stressed, or infected cells and healthy tissues. Such materials were typically based on hydrogels, whose elastic modulus falls within the physiological range of the T-cell environment, such as target cells or APCs, which exhibit cortical Young’s moduli from approximately hundreds of pascals to tens of kilopascals. Importantly, while hydrogels are not defined as nanomaterials per-se, their relevance here is not simply due to their material composition, but mostly due to their ability to present or control cellular cues at the nanoscale. Thus, they provide a precursor material platforms that established design principles later implemented in nanostructured systems.

Indeed, the tunable elasticity of activating materials has been shown to be an important regulator of T-cell responses. One widely used hydrogel system with controllable elasticity is based on polyacrylamide. Judokusumo et al. varied the ratio between the acrylamide precursor and bis-acrylamide crosslinker to tune the elastic modulus of the resulting hydrogel over a range of approximately 10 Pa to 200 kPa, demonstrating that increasing substrate stiffness enhances T-cell activation and early signaling (90). Later, Saitakis et al. showed that increasing the modulus of acrylamide-based activating materials within a similar range not only enhances T-cell activation, but also modulates cell morphology, motility, and metabolism (71). A substantially broader stiffness range, extending up to several MPa, was achieved using polydimethylsiloxane (PDMS). Within this higher-modulus regime, the activation–stiffness relationship was found to invert (70), suggesting that the response of T cells to the stiffness of ligand-presenting surfaces is not linear, but rather biphasic, with maximal activation occurring at an intermediate stiffness within the physiologically relevant range. This biphasic behavior was subsequently confirmed by high-resolution probing of T-cell responses across finely tuned polyacrylamide moduli (85). A similar trend was also observed for T-cell spreading over a broad range of PDMS stiffnesses, from approximately 0.5 kPa to several MPa (86).

In parallel, modulating stiffness beyond the physiological range was explored as a strategy to enhance ex vivo T-cell activation and proliferation. In this context, not only flat surfaces (70) but also microstructures based on elastomers were shown to significantly affect activation and proliferation, paving the way for using stiffness as a design parameter in engineered activation platforms. Lamber et al. used elastic PDMS microbeads as an alternative to rigid Dynabeads, for mechanoistimulative T cell activation (91). Similarly, Dang et al. engineered electrospun poly(ϵ-caprolactone) fibers presenting CD3/CD28 ligands, softened by incorporation of PDMS, to enhance expansion of human CD4+ and CD8+ T cells and restore proliferative responses in otherwise poorly responsive T cells from CLL patients (92). Finally, elastic gels can tune T cell response beyond which is beyond outcomes. Recently, polyethylene glycol diacrylate (PEGDA) hydrogel scaffold with tunable elasticity was shown drive T cell phenotypic profiling, particularly focusing on T stem cell memory T_SCM_ and central memory T_CM_ phenotypes that are critical for sustained cancer killing (93).

### 2.4 Nanostructured materials for topographic and curvature-mediated T cell stimulation

T cells respond to ex vivo activating surface microtopography by altering cytoskeletal dynamics, immune synapse formation, signaling, and activation outcomes, often mimicking APC interfaces. Such microtopography can be induced by elastic micropillars, which can be produce by casting PDMS on 3D micropatterned molding surface. Originally used as a tool in mechanobiological studies (94–96), they were later adapted for T cell studies, It was shown in particular, that T cells deform structures via contractility, delaying MTOC centralization on stiffer pillars (97), and inducing enhanced activation and proliferation compared to flat surfaces of the same modulus (98). Similarly, microscale topography ban be produced by PDMS micro-pits, which induce the formation of actin-rich protrusions, with a biphasic effect on T-cell activation depending on the dimensions of the microtopographic features (99).

While PDMS-replicated structures provide a simple and robust platform for investigating the effects of topography at the microscale, extending this approach to the nanoscale is considerably more challenging. Submicron features are difficult to reproduce by conventional PDMS molding because the relatively high viscosity of the precursor limits its ability to infiltrate very small structures. To overcome this limitation, bottom-up approaches can be employed to generate nanoscale materials. Nanowires, which are high-aspect-ratio nanostructures that can be synthesized by methods such as chemical vapor deposition, provide an attractive platform for probing cell–material mechanical interactions at the nanoscale (100). We (MS) used synthetic zinc oxide and silicon nanowire arrays to demonstrate that dense nanoscale topographies enhance lymphocyte activation, first in NK cells (101–103) and subsequently in T cells (77). In these systems, activation is governed not only by the nanoscale topography of the nanowire forest, but also by nanowire length, which determines the flexibility of individual nanowires and the collective mechanical response of the array to cell-generated forces. These findings therefore highlight the coupled effects of topography and elasticity of the activating surface on T-cell responses.

Importantly, in all these studies, the topographic surfaces were functionalized with activating ligands, sometimes combined with costimulatory ligands (usually anti-CD3 and anti-CD28), to provide the biochemical signals essential for T-cell activation. Thus, these works present combined chemical–topographic stimuli. However, topographic stimuli per se can activate T cells to a certain extent. Non-functionalized nanowires induce higher T-cell activation than non-functionalized flat surfaces, and this activation gradually increases with nanowire length (i.e., with decreasing nanowire mechanical resistance) (77).

The observed levels of activation, although smaller compared to those obtained on nanowires coated with ligands, cannot be attributed to tonic signaling and are likely the result of high membrane curvature at the cell–nanowire interface. This curvature can induce segregation between TCR and CD45 phosphatase (72, 76), thereby decreasing inhibition of TCR signaling by CD45 (104, 105).

A similar effect of induced membrane curvature was demonstrated through increased activation and expansion of T cells using anti-CD3/anti-CD28-coated Au nanoparticles with spiky surface topography (106). Similarly, arrays of nanopores of anodized Aluminum Oxide without ligand functionalization induce the formation of microvilli and show increased T-cell activation, particularly for ~200 nm pores (78). These studies demonstrate pore size-dependent exclusion of CD45 phosphatase from TCR at microvilli tips, confirming that a non-negligible (compared to tonic) activation level can be achieved solely by inducing appropriate membrane curvature.

### 2.5 Nanopatterned materials for controlled ligand distribution and receptor clustering

While unbound receptors and ligands, as well as ligand–receptor pairs, are organized into highly regulated clusters whose shape and distribution change during different stages of T-cell–target interaction (107), this clustering can be mimicked using artificial antigen-presenting surfaces patterned with ligands. These ligands can be arranged using a variety of patterning methods and across multiple length scales. However, such systems differ from the *in vivo* stimulating environment in that they provide a static and spatially controlled distribution of ligands.

Early work in this area involved patterning microscale domains of activating ligands surrounded by adhesion ligands—a configuration that partially mimics the structure of a mature immune synapse—and examining how this spatial organization affects T-cell responses (108). Such patterns were generated by photolithographic positioning of anti-CD3 microdomains, which engage the TCR/CD3 complex, within surrounding regions of ICAM, a ligand for T-cell integrins. However, conventional photolithography is limited in spatial resolution and typically cannot generate features substantially below the micrometer scale using standard laboratory equipment. To overcome this limitation, later advances in nanofabrication enabled ligand organization at the nanometer scale. A prominent example is the tethering of ligands to arrays of metallic nanodots produced by block-copolymer lithography (109). However, this approach is largely restricted to continuous, periodic arrays and therefore does not readily allow independent discrimination between the effects of total ligand surface density and local clustering. This limitation was subsequently addressed using high-throughput nanoimprint lithography, which enables the fabrication of ligand-functionalized metallic nanodots arranged in arbitrary geometries (110). In these systems, sub-10-nm nanodot dimensions enable control over ligand organization approaching the single-molecule level (111).

This approach has been applied to study the effect of antigen molecule distribution on lymphocyte activation, including T cells. Continuous ligand arrays with varied inter-ligand spacing, which were produced by block copolymer lithography or nanoimprint, were used to establish the optimal distribution of ligands for activation (82, 83, 85, 112). Yet, due to the continuous ligand arrangement in there arrays, it was unclear whether the activation was governed by the spacing between the ligands, or their overall surface density, and these two parameters were interdependent. Later, arrays of nanodots with arbitrary arrangement, produced by electron beam lithography and functionalized with ligands, enabled to reveal that localized clustering is a more dominant factor in determining T-cell response than overall ligand density (84).

The effect of ligand clustering on T-cell activation has been rationalized using theoretical models based on cooperative receptor–ligand binding. Formation of an initial receptor–ligand bond locally suppresses membrane fluctuations, thereby increasing the probability that neighboring receptors will bind nearby ligands. Each newly formed bond further stabilizes the membrane and promotes additional bond formation, creating a positive-feedback process that continues until the local cluster approaches saturation. This mechanism was first demonstrated for NK cells (113) and was later supported by experiments with T cells interacting with arbitrarily patterned ligand arrays (86). Together, these studies showed that even at very low global ligand densities, robust T-cell activation can occur when ligands are concentrated into dense nanoclusters, where neighboring receptor–ligand pairs cooperatively influence one another’s binding and unbinding kinetics.

### 2.6 Multifunctional materials with tuned physical cues for CAR T-cell generation

Insights into the mechanisms of T-cell responses to physical cues provided by nanoengineered ex vivo activating surfaces (**Figure 3**), together with advances in fabrication methods, have naturally led to the development of next-generation platforms designed not only to study T-cell activation mechanisms but also to engineer controlled T-cell priming platforms for adoptive immunotherapy, which can enhance the functional outcomes of immunotherapeutic T cells. However, the adoption of such platforms in CAR T-cell immunotherapy has been relatively limited so far. This section discusses several recently published nanomaterial-based T-cell priming platforms that have been shown to improve the immunotherapeutic outcomes of CAR T cells. While presentation of activating and costimulatory cues can be achieved using a broad range of nanoscale materials onto which anti-CD3 and anti-CD28 antibodies are immobilized, synthesizing nanomaterials that combine these cues with soluble factors can significantly facilitate the CAR T-cell generation process.

**Figure 3:**
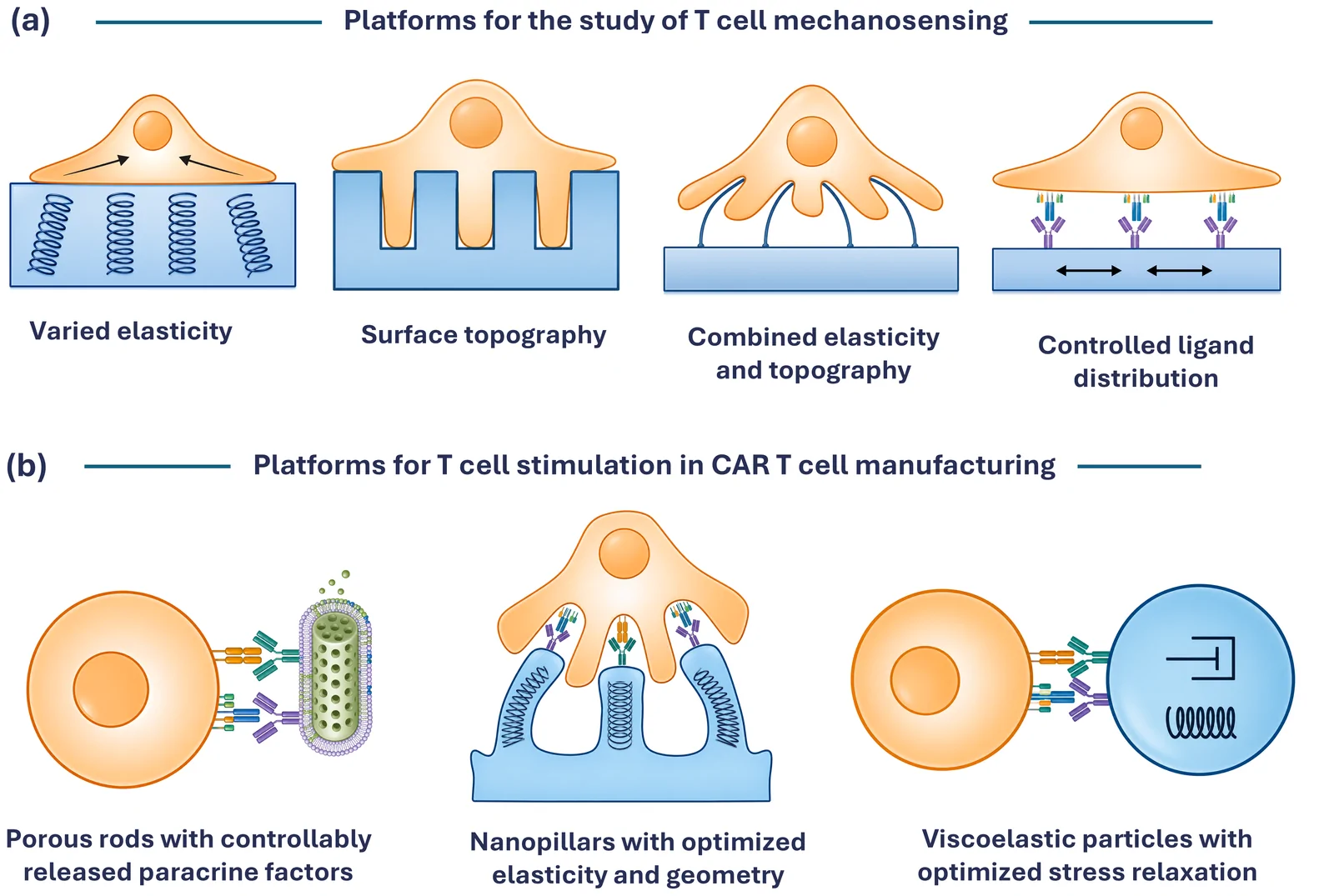
Artificial nanostructured platforms with controllably introduced physical cues, used for the fundamental study of T cell activation mechanism, as well as for tunable activation in the workflow of CAR T-cell manufacturing.

Recently, Mooney’s group synthesized inorganic mesoporous silica nanorods coated with a lipid bilayer, to which anti-CD3 and anti-CD28 were tethered, while the mesoporous silica rods were loaded with paracrine factors that were controllably released upon introduction of the material into T-cell culture. The prominent feature of these rods is that in cell culture, they form 3D scaffolds, which are infiltrated by T cells, allowing effective rod-cell interfacing.This material greatly increased polyclonal expansion of primary mouse and human T cells compared to Dynabeads and was particularly effective in restimulating CAR T cells (43).

In follow-up work, these nanorods were used to activate primary human T cells as the first step of the CAR T cell production process (44). The advantage of these nanorods over Dynabeads lies in the ease of controlling the stimulation dose, which was found to be critical for the immunotherapeutic outcome of CAR T cells. Specifically, CAR T cells were generated from primary T cells of healthy donors, as well as from patients with acute lymphoblastic leukemia (ALL) and chronic lymphocytic leukemia (CLL), and a correlation between stimulation dose, T-cell phenotype, and antitumor activity (in murine models) was observed in a patient-specific manner. Interestingly, this work proposes the use of T-cell-activating nanomaterials in a way that enables patient-specific control over the overall stimulation dose, opening a pathway toward more personalized CAR T immunotherapy, in which the stimulation step can be tailored rather than standardized.

Alternative T-cell stimulating materials are based on insights gained from studies in which materials were designed to mimic the physical cues presented by natural T-cell activating surfaces. Being inspired by previously reported enhancement of T-cell activation by mixed mechanical cues presented in ligand-functionalized nanowires (77), we showed that a similar enhancement effect can be achieved by replacing inorganic nanowires with elastomeric pillars made of polydimethylsiloxane (PDMS), whose geometry is designed to produce similar mechanical resistance to T-cell forces and to induce nanoscale curvature of the T-cell membrane, as nanowires do (98).

We next employed these nanopillars in CAR T-cell production (45). Based on previous reports on topography-dependent (77, 78) and modulus-dependent (70, 71, 90) T-cell activation, we hypothesized that independent variation in the topography and modulus of the ligand-supporting surface would determine not only activation and proliferation levels but also other response functions essential for CAR T-cell therapeutic efficacy, including exhaustion, CAR transduction efficiency, and the prevalence of the central memory phenotype in the proliferated CAR T-cell population. We therefore generated a library of stimulating surfaces with varying moduli and pillar topographies and used it to produce CAR T cells from primary T cells of multiple donors, followed by analysis of different functional outcomes. Using multivariate analysis, we identified a design of the stimulating surface that provides the best combination of high activation, high proliferation, low exhaustion, and high transduction efficiency.

We demonstrated the advantage of this stimulating platform over Dynabeads through an increased population of central memory phenotype within the produced CAR T cells, as well as improved antitumor efficacy in both ex vivo and *in vivo* cancer killing assays. Remarkably, these best-performing pillars were of submicron dimensions, whose fabrication required relatively expensive and slow processes (i.e., electron-beam lithography) adopted from semiconductor device technology. However, we recently demonstrated that such a stimulating platform can be made scalable through a novel self-assembly-based patterning approach and can be readily produced at a physical scale sufficient for generating CAR T cells in quantities required for clinical treatment (46).

### 2.7 Viscoelastic materials for the effective CAR T cell generation

Mechanical properties of the ligand-supporting material can be further tuned to improve CAR T-cell generation. Importantly, the elastic modulus of the ligand-carrying material has traditionally been the primary parameter modified to study or tune ex vivo T-cell responses. However, the natural T-cell environment—including antigen-presenting cells and target cells—is viscoelastic. In simplified terms, viscoelasticity refers to a material’s time-dependent response to mechanical stress, exhibiting either creep under constant load or stress relaxation under constant deformation. In recent years, substantial efforts have been made to design materials with independently tunable elastic and viscous components, enabling multiparametric mechanical control of cellular responses (114).

Recently, a model extracellular matrix with decoupled elastic and viscous components was used to demonstrate that environmental viscoelasticity regulates T-cell responses and phenotype composition (57). In the context of CAR T cells, a seminal work by Liu et al. introduced alginate-based viscoelastic particles that mimic the viscoelasticity of APCs (47). Using a microfluidic platform, the authors fabricated alginate-based microparticles in which viscoelasticity is controlled via ionic crosslinking of low–molecular weight alginate (resulting in fast stress relaxation), while purely elastic counterparts are generated through covalent crosslinking of higher–molecular weight alginate (resulting in minimal stress relaxation). This design enabled decoupling of elastic stiffness (in the kPa range) from stress relaxation timescales, allowing systematic investigation of their distinct roles in T-cell activation. Functionally, while variations in stiffness alone had modest effects, stress relaxation (i.e., viscoelasticity) emerged as the dominant regulator of T-cell fate. Viscoelastic SynVACs enhanced immunological synapse formation, increased T-cell expansion, drove CD8^+^ skewing, and significantly enriched stem-like memory T cells (T_SCM_), while reducing regulatory T-cell formation. In contrast, elastic (non-relaxing) beads—even at similar stiffness—failed to reproduce these effects, highlighting that time-dependent mechanical cues, rather than static elasticity, govern T-cell programming. These phenotypic advantages translated into approximately 90% CAR transduction efficiency, enhanced cytotoxicity, and superior *in vivo* persistence and tumor control. Overall, this work established stress relaxation as a key mechanobiological parameter for T-cell activation in CAR T-cell engineering, which should be taken into account in the design of future CAR T-cell production platforms.

## 3 Genetic reprogramming of T Cells with CAR

### 3.1 Reprogramming with CAR – state of the art and its limitations

In standardized CAR T-cell manufacturing, the transduction step involves exposing activated T cells to a replication-incompetent viral vector, most commonly a lentiviral or gamma-retroviral vector, that carries the CAR transgene, allowing stable genomic integration and sustained CAR expression after entry into the cell (115). This approach is favored because activated T cells are highly permissive to viral gene transfer, and viral delivery generally provides high transduction efficiency, robust expression, and compatibility with both proliferating and non-dividing cells. Its main advantages are durable CAR expression and well-established clinical manufacturing workflows.

On the other hand, its principal limitations include high production cost, process complexity, payload constraints, and safety concerns such as insertional mutagenesis, immunogenicity, and the possibility that overly strong CAR expression can contribute to tonic signaling and T-cell exhaustion.

Viral and non-viral gene-delivery approaches therefore present distinct manufacturing tradeoffs. Lentiviral and gamma-retroviral vectors remain the most clinically mature platforms because they provide relatively efficient and durable CAR expression, but vector production is costly and complex, and integrating vectors carry a low but non-zero risk of insertional genotoxicity. Non-viral approaches can simplify manufacturing and avoid viral-vector production; however, transient mRNA delivery may require repeat administration, whereas DNA-, transposon-, and genome-editing-based approaches still face challenges in delivery efficiency, genotoxicity or off-target editing, and clinical standardization (116). Thus, viral vectors currently retain an advantage in clinical readiness, while non-viral systems may offer future gains in scalability, flexibility, and manufacturing cost.

In light of these constraints, transduction efficiency—defined as the percentage of CAR-expressing T cells after transduction—is naturally considered one of the key criteria for the success of the entire production cycle. Typical CAR T-cell viral transduction efficiencies are often reported in the range of approximately 30% to 70% in clinical manufacturing, although the exact rate varies widely depending on the vector, CAR construct, and cell source (115). The efficiency is mainly determined by multiplicity of infection, the activation state and timing of the T cells, vector titer and stability, promoter and CAR design, and culture conditions such as transduction enhancers and temperature. Furthermore, transduction efficiency varies across donors. Therefore, improving transduction efficiency and minimizing its variability across different donors is therefore an important aspect of CAR T-cell production that can be facilitated by nanoscale materials and systems rationally designed for this purpose.

### 3.2 Nanotechnology for improved viral transduction

While most advances in this field focus on designing nanomaterial-based approaches to bypass viral transduction, several reports demonstrate how nanomaterials can improve virus-based CAR T-cell production. Aramesh et al. recently reported the use of a polycaprolactone-based nanoporous substrate to improve lentiviral uptake by T cells (48). Interestingly, transduction efficiency was found to improve in a size-dependent manner, with the highest efficiency observed for ~500 nm pores. The resulting CAR T cells exhibited higher cytotoxicity in ex vivo cancer killing assays. The authors proposed that the nanopore topography enhances macropinocytosis, thereby increasing viral uptake.

An additional approach is based on glycometabolic bioorthogonal chemistry-guided viral transduction (49). In this method, T cells are treated with unnatural sugars (e.g., Ac4GalNAz) to incorporate azide groups onto their surface. DBCO-functionalized lentiviruses then bind specifically to the azide-labeled T cells via azide–alkyne cycloaddition. CD19-targeting CAR T cells produced using this method showed improved antitumor efficacy in B-cell lymphoma xenograft mouse models, with enhanced cell proliferation, infiltration, and cytokine secretion.

### 3.3 Non-viral programming with CAR

Still, most works using nanomaterials for improving CAR T-cell engineering are aimed at alternatives to viral transduction, such as mRNA transfection. mRNA electroporation is rapid (manufacturing in 1–2 days), avoids insertional mutagenesis and complex viral production, allows easy re-dosing and rapid CAR prototyping, and can be tuned using modified nucleosides, cap structures, and newer circular mRNA (cmRNA) platforms that significantly prolong CAR expression and enhance antitumor activity compared to linear mRNA (117). However, because expression is short-lived (typically peaking within 6–24 h and diminishing within ~3–7 days even with optimized constructs), repeated infusions are required and *in vivo* persistence is limited (118, 119), and, overall efficacy for many indications still lags behind virally transduced CAR T cells, which can persist for years and mediate durable remissions (120, 121).

As of now, there are no FDA-approved CAR T therapies based on mRNA transfection; all approved products rely on viral vectors. Nonetheless, mRNA-engineered CAR T cells are being evaluated in multiple early-phase clinical trials—for example, mesothelin-targeted RNA CAR T cells in pancreatic cancer (122), cMET RNA CAR T cells in metastatic breast cancer and melanoma, among others—amounting to approximately 10–20 registered mRNA CAR T trials globally, primarily phase I safety and feasibility studies. Still, despite its promise, standard cuvette-based electroporation still faces technical challenges, including a trade-off between transfection efficiency and viability (often ~40–70% viability with 60–80% CAR^+^ cells), non-uniform electric fields leading to heterogeneous mRNA uptake, and scalability limitations.

In recent years, several nanotechnological platforms have been explored to address these challenges and advance mRNA-based CAR T-cell production. Mitchell group explored ex vivo delivery of mRNA to human T cells using lipid nanoparticles (50) (schematically shown in **Figure 4a**). They synthesized a library of ionizable lipid-based nanoparticles and selected a formulation with optimal luciferase mRNA delivery to Jurkat cells. This formulation was subsequently used for CAR mRNA delivery to primary human T cells, resulting in CAR expression at levels comparable to electroporation, but with significantly reduced cytotoxicity. The produced CAR T cells were effective in cancer killing assays against Nalm-6 acute lymphoblastic leukemia cells, at least at the level of CAR T cells generated via electroporation-based mRNA delivery.

**Figure 4:**
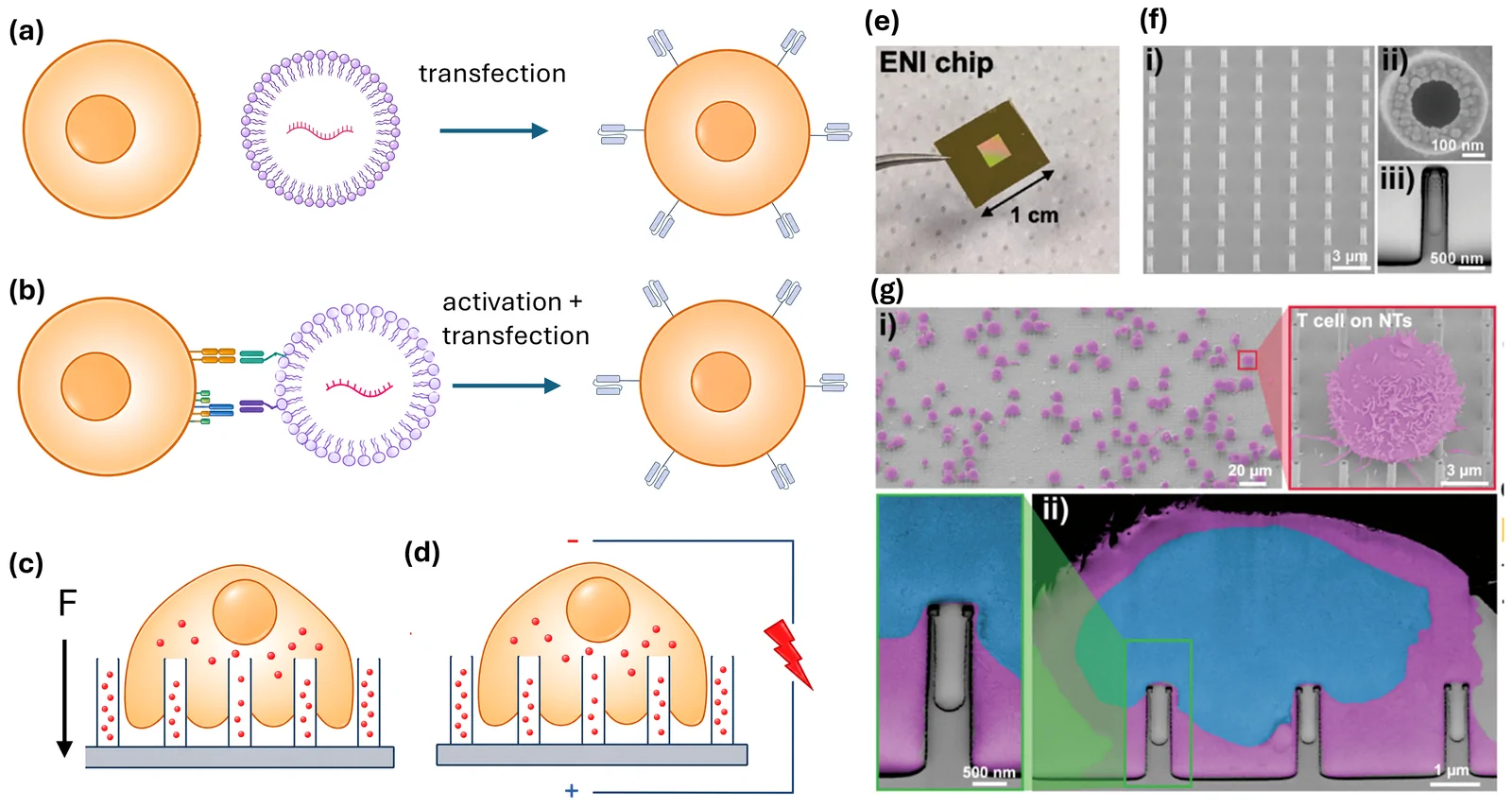
Various nanotechnological approaches for mRNA delivery to produce CAR T cells. **(a)** mRNA encapsulated lipid nanoparticle, **(b)** mRNA encapsulated in lipid nanoparticles with surface-immobilized activating and costimulatory ligands for simultaneous specific targeting and stimulation of T cells, **(c)** Nanostraws for passive delivery, **(d)** Nanostraws for delivery facilitated by electric field. **(e, f)** Photographic image and SEM, respectively, of a chip containing the nanostraw based delivery platform, **(g)** False-colored SEM images showing the interfacial interactions between primary human T cells and Au-coated NTs arrays **(i)** and the cross-sectional profile of nanostraw–membrane interface (ii). **(e–g)** reproduced with permission from ref (54).

This seminal work, which opened a new perspective for CAR T-cell production, served as a foundation for several follow-up studies by the same group. In these studies, the mRNA-delivering lipid nanoparticles were modified with additional functionalities to improve CAR T-cell immunotherapy. First, nanoparticle formulations were optimized by refining the molecular ratios of ionizable lipids, cholesterol, phospholipids, and lipid-anchored PEG, leading to improved mRNA delivery (51). Further development combined CAR immunotherapy with immune checkpoint knockdown by co-encapsulating CAR mRNA and small interfering RNA (siRNA) targeting PD-1, resulting in CAR T cells with transient CAR expression and temporary cell-intrinsic PD-1 disruption (129).

In another modification, the surface of the mRNA-encapsulating lipid nanoparticles was functionalized with activating and costimulatory antibodies (52) (**Figure 4b**). This design combines two key functionalities: engagement of activating and costimulatory receptors, which is an essential step in CAR T-cell production, and delivery of CAR-encoding genetic material. Therefore, it enables skipping the initial activation stage with antibody-coated beads, simplifying and shortening the production cycle.

Interestingly, a similar nanoparticle design was shown to be applicable for the production of CAR monocytes (130). Finally, this concept of CAR-encoding mRNA delivery was extended toward *in vivo* CAR T-cell generation, which is discussed later in this review. While mRNA delivery using nanoparticles is among the most promising approaches for future CAR T-cell production, alternative strategies have also been explored in recent years. One-dimensional nanoscale materials, such as nanowires and nanoneedles (131, 132), as well as hollow structures such as nanostraws (133–138), have long been investigated as effective platforms for intracellular delivery of genetic materials. Naturally, with the emergence of CAR T-cell immunotherapy, these nanotechnological approaches have been applied, among other applications, to non-viral CAR T-cell generation. Beyond transient CAR expression, these intracellular delivery platforms may also facilitate future CRISPR/Cas-mediated genome engineering approaches for CAR T-cell manufacturing (139). Efficient nanoscale delivery of gene-editing components could enable multiplexed modifications, including targeted CAR knock-in, disruption of inhibitory receptors, elimination of endogenous TCR expression, and engineering of additional functional attributes without reliance on viral vectors (140, 141).

A remarkable and pioneering work by the Voelker group introduced an mRNA transfection platform based on fabricated silicon nanotube arrays loaded with PCR expression cassettes encoding an anti-CD19 CAR (53) (Schematically shown in **Figure 4c**). CAR T cells seeded on these arrays exhibited effective CAR transduction, which was confirmed by successful cancer killing assays. Interestingly, the nanotube platform is considered a passive delivery system, meaning that no external forces are required to enable intracellular delivery of genetic material. Instead, delivery occurs through tight membrane invagination around the nanotubes, which generates localized stress and membrane permeabilization. However, this stress and membrane permeabilization reduced cell viability for arrays with high nanotube density.

Alternatively, external forces can be applied to enhance transfection efficiency. The seminal work of Shokouhi et al. demonstrated that Silicon nanotube platform can be couples with electrical stimulation by applying localized electric fields in pulses of 10 V, resulting in transient pore formation at the interface between the cell membrane and nanotubes (54) (**Figures 4d–g**). These pores enabled influx of the PCR expression cassette stored within the nanotubes, achieving cell viability above 90% and transfection efficiency of approximately 70%, which is about twice as high as previously reported for passive nanowire-based delivery systems.

## 4 Ex-vivo expansion of CAR T cells

Ex vivo expansion is the final manufacturing stage in conventional CAR T-cell production, in which engineered cells are cultured under controlled conditions until they reach a therapeutic dose for infusion. Clinically, this step is critical because it determines whether the product contains sufficient viable and potent cells for reinfusion and strongly influences the final phenotype, persistence, and antitumor activity of the CAR T-cell product. In practice, expansion is typically carried out over approximately 1–2 weeks, with many protocols using 9–14 days of culture, although some workflows extend longer depending on the platform. The culture is maintained under tightly controlled cell density and GMP conditions, with the goal of expanding the engineered T-cell population to a final clinical dose, commonly on the order of hundreds of millions to billions of CAR T cells.

The main challenges of this stage include preserving cell fitness while achieving large-scale expansion, as prolonged culture can drive differentiation, loss of stem-like features, reduced proliferative capacity, and diminished *in vivo* persistence. In addition, this step is expensive, time-consuming, highly variable between patients, and constrained by sterility, logistics, and release testing requirements.

In current clinical CAR T-cell manufacturing, cells are most commonly expanded ex vivo in suspension culture under GMP conditions, using closed or semi-closed bioreactors or gas-permeable bag systems, rather than on adherent 2D surfaces. The basic environment consists of a 37 °C, 5% CO_2_ humidified incubator with tightly controlled pH, osmolarity, and nutrient supply, typically using serum-free or serum-supplemented T-cell media supplemented with cytokines such as IL-2. In practical terms, this corresponds to a physiologically relevant, nutrient-rich, gas-controlled liquid culture environment designed to preserve viability and expansion efficiency while remaining compatible with clinical manufacturing standards. However, suspension culture remains a simplified surrogate of the *in vivo* lymphoid niche, and the chosen media and cytokine conditions can bias differentiation state, proliferation rate, and exhaustion risk.

In this context, there has been a growing effort to engineer artificial environments for CAR T-cell proliferation, aiming to better mimic the lymphoid niche and, specifically, its mechanical and structural characteristics, thereby steering CAR T-cell proliferation toward more potent immunotherapeutic products. One of the key characteristics of such engineered niches is their mechanical properties, which have been shown in several studies to modulate CAR T-cell proliferation and phenotype in a controlled manner.

Hydrogels with tunable composition and stiffness are among the most commonly used approaches for such controlled proliferation. For example, Castellote-Borrell et al. engineered a hydrogel based on a poly(ethylene glycol)–heparin mixture, whose structure included interconnected micropores (55). Optimization of pore size and stiffness enabled improved CAR T-cell expression and expansion, achieving approximately a twofold increase in replication index compared to suspension cultures.

Similarly, Jie et al. used a gel based on self-assembling peptides, which enabled shortening of proliferation time while achieving approximately a 10-fold increase in CAR T-cell expansion compared to traditional protocols, under optimized matrix stiffness conditions (56). Further improvements in proliferation were achieved by engineering CAR T cells that secrete intrinsic PD-1 scFv, which also resulted in increased *in vivo* antitumor activity in murine models.

Not only the elastic component, but also the viscous component of the CAR T-cell expansion niche can influence proliferation and cell fate. Adu-Berchie et al. recently reported a collagen-based CAR T-cell niche with independently controllable elasticity and stress relaxation (57) This system was used to expand T cells following standard CD3/CD28 bead activation, and it was found that matrix stress relaxation is a dominant driver of the resulting phenotype. Specifically, slow-relaxing matrices promoted a more activated, effector-like state characterized by higher AP-1 signaling, increased expression of inhibitory markers, and reduced expression of memory markers, whereas fast-relaxing matrices preserved a more memory-like phenotype. In CAR T-cell experiments, cells cultured in slow-relaxing matrices exhibited stronger *in vitro* cytotoxicity and improved *in vivo* tumor control in a Raji xenograft model under certain stimulation conditions. However, chronic stimulation in the same slow-relaxing environment could exacerbate dysfunction, highlighting the interplay between mechanical context and stimulation history. Importantly, the observed effects were attributed to direct phenotypic reprogramming rather than clonal selection. From a practical perspective, this work suggests that future bioprocesses could be tuned to bias CAR T-cell products toward either memory-like, persistent phenotypes or highly cytotoxic effector phenotypes by adjusting matrix mechanical properties during expansion. It also highlights a pathway toward more biomimetic culture systems, while leaving open important challenges related to scalability, GMP compatibility, and translation into clinical workflows.

One of the key components of the phenotypic profile of CAR T cells relevant for immunotherapy is the presence of T_SCM_ cells. This subset uniquely combines the self-renewal capacity of stem cells with the functional potential of effector T cells (15, 142, 143). This makes of T_SCM_ cells fundamentally different from more differentiated CAR T-cell populations and highly desirable for durable immunotherapy, as they maintain long-term persistence, exhibit low exhaustion, enable sustained immune surveillance, and generate multiple downstream T-cell subsets (central memory, effector memory, and cytotoxic effector cells), effectively acting as a reservoir that continuously replenishes functional CAR T cells. These cells are typically characterized by markers such as CCR7^+^, CD45RO^−^/^+^, CD95^+^, and expression of stemness-associated genes (e.g., TCF7, LEF1), and they exhibit transcriptional programs associated with longevity and proliferative capacity.

In this context, a recent study by Lv et al. demonstrated that a gel-based niche can decouple differentiation from proliferation of CAR T cells and specifically tune its mechanical properties to enrich the population of stem-like CAR T cells (144). In this work, the ex vivo expansion niche was engineered using a fibrin-based gel. Expansion of CAR T cells produced via anti-CD3/CD28-functionalized bead activation and lentiviral transduction resulted in conversion of differentiated T cells into a stem-like phenotype. Mechanistically, the soft 3D fibrin environment engages β2 integrin, which recruits 14-3-3ζ to phosphorylate and sequester YAP in the cytoplasm, thereby derepressing the MafG transcription factor and activating stemness-associated genes. Notably, CAR T cells in fibrin gels spontaneously form spheroids, mimicking natural tissue niches and amplifying mechanical signaling to sustain stemness without inducing exhaustion. These stem-like CAR T cells demonstrated superior *in vitro* cytotoxicity and improved *in vivo* tumor control in solid tumor models compared to conventionally expanded 2D controls.

## 5 Nanotechnology for *in vivo* CAR T-cell manufacturing

The motivation to develop *in vivo* CAR T-cell production arises from the fundamental limitations of current ex vivo manufacturing, which is complex, costly, time-consuming, and patient-specific. Conventional workflows include leukapheresis, genetic modification, expansion under GMP conditions, and reinfusion—processes that can delay treatment, particularly for rapidly progressing malignancies. The overall cost of CAR T-cell therapy can exceed US$500,000 per patient, substantially limiting accessibility and global implementation.

*In vivo* approaches aim to bypass these constraints by directly delivering gene-engineering components (e.g., viral vectors or nanoparticle systems) to T cells within the patient, enabling *in situ* generation of functional CAR T cells. However, this paradigm introduces significant challenges, including achieving selective and efficient targeting of T cells in a complex biological environment, controlling transduction levels to ensure safety and efficacy, avoiding off-target gene delivery, and mitigating immune responses against vectors or engineered cells. Additional challenges include regulating activation signals to prevent exhaustion or cytokine release syndrome, as well as ensuring reproducibility and scalability comparable to controlled ex vivo systems.

Nanoscale materials offer a powerful route to enable *in vivo* CAR T-cell production by acting as programmable carriers that integrate three critical functions: (i) targeting, (ii) activation, and (iii) gene delivery, within a single platform (**Figure 5**). The first two functions are often achieved by functionalizing the surface of the platform with ligands (e.g., anti-CD3 or anti-CD8 antibodies) that selectively bind circulating T cells, thereby increasing the specificity of gene transfer in the bloodstream or lymphoid tissues. The third function is achieved through encapsulation or conjugation of the genetic cargo (mRNA, DNA, or viral vectors), protecting it from degradation while enabling controlled intracellular release, thereby improving transduction efficiency without the need for ex vivo manipulation.

**Figure 5:**
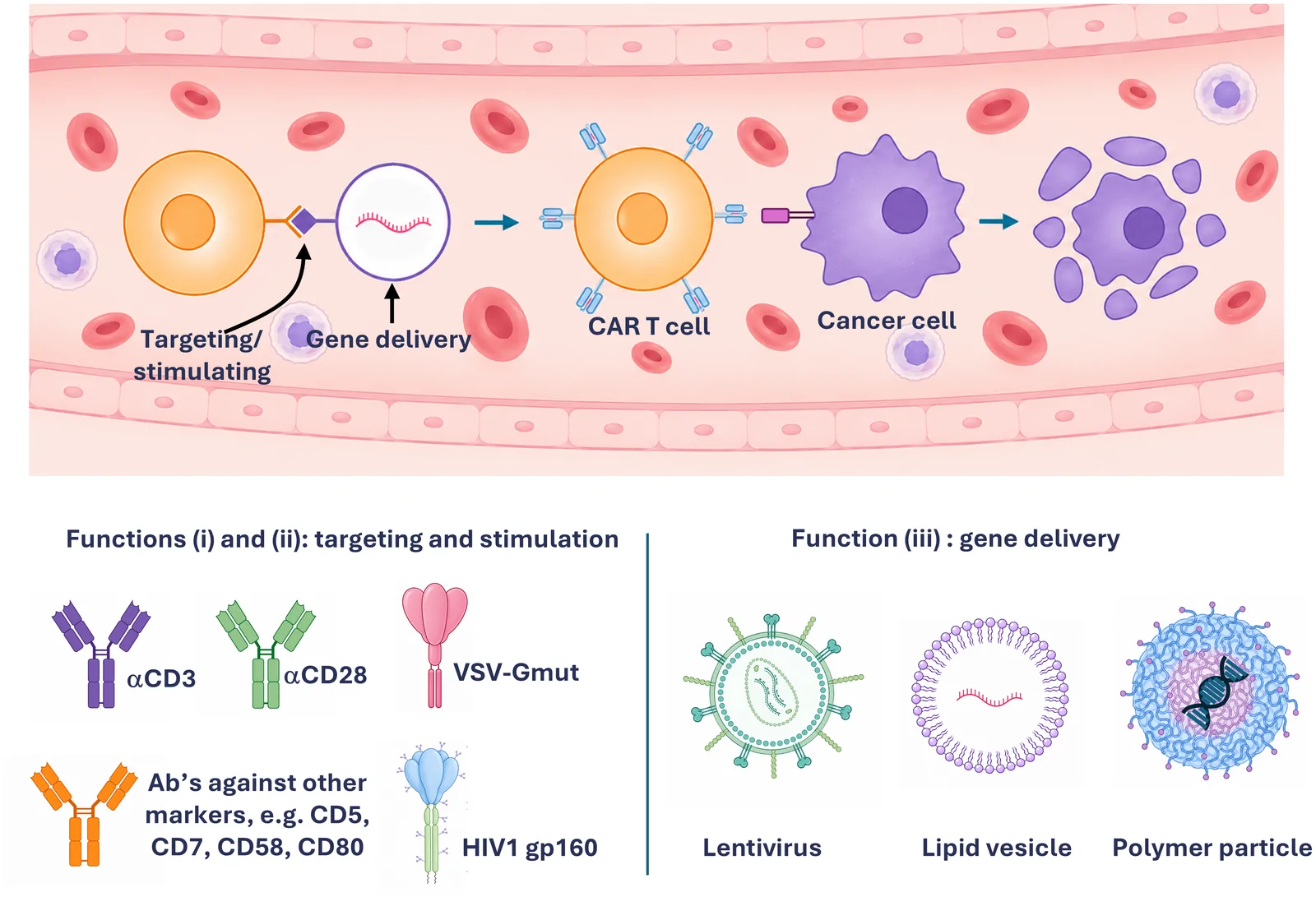
Scheme of *in-vivo* CAR T-cell generation, and variable key components for the *in-vivo* engineering platforms.

Beyond these core functions, nanoscale design allows precise control over physical and biochemical parameters such as particle size, shape, stiffness, and surface chemistry, which can influence cellular uptake, trafficking, and even mechanotransduction pathways that regulate T-cell activation and differentiation. **Table 1** presents examples of *in vivo* approaches currently being explored in academia and industry, categorized by delivery technology and stage of development.

An early example of such nanoparticles is based on biodegradable poly(β-amino ester) (PBAE) polymers functionalized with MTAS-NLS peptides for nuclear import, surface-coated with polyglutamic acid (PGA)–anti-CD3ϵ F(ab’)_2_ conjugates for selective T-cell binding and endocytosis, and loaded with piggyBac transposon plasmids encoding a CD19-specific 19-41BBz CAR together with a hyperactive transposase for stable genomic integration (145). These particles, approximately 150 nm in size, were stabilized by a negative zeta potential and exhibited lyophilization compatibility for off-the-shelf use. They showed low off-target uptake and up to 20% transfection efficiency. In immunocompetent mice with B-cell acute lymphoblastic leukemia, five intravenous doses reprogrammed host T cells to proliferate, persist as memory cells, and eradicate tumors with efficacy comparable to ex vivo CAR T-cell infusions, while causing minimal toxicity or cytokine release.

Another recent example using PBAE-based materials for *in vivo* CAR T-cell generation involves mRNA-delivering nanoparticles constructed from a lipophilic–hydrophilic PBAE core, PEG-lipid insertion, and surface conjugation of anti-CD3 and anti-CD28 antibodies (146). These nanoparticles efficiently encapsulate CAR mRNA, exhibit a size of approximately 200 nm with near-neutral surface charge after formulation, and outperform untargeted particles and even a Moderna-style lipid nanoparticle comparator in T-cell transfection. *In vitro*, these dual-ligand nanoparticles drive strong CAR expression, T-cell activation, memory-like phenotype, and proliferation comparable to Dynabeads. *In vivo*, they redirect transfection toward the spleen and lymph nodes while reducing uptake by the liver and macrophages. Most importantly, systemic administration of anti-CD19 CAR mRNA nanoparticles induces potent, transient CAR T-cell generation, resulting in deep B-cell depletion in blood and spleen, including up to approximately 95% depletion in blood after a single dose and strong effects upon repeated dosing.

Lipid nanoparticles (**Box 2**) represent another intensively explored approach for *in vivo* CAR T-cell generation. Following the development of such nanoparticles for the ex-vivo mRNA delivery to T cells (51), Mitchel group extended this concept to *in vivo* CAR T-cell generation (147). In this work, the selected C14–4 lipid was used as the basis for mRNA-delivering nanoparticles, which were further modified using maleimide-functionalized polyethylene glycol (PEG) lipid to conjugate antibodies such as anti-CD3, anti-CD5, and anti-CD7 for selective targeting of T cells. This platform enabled transient mRNA delivery of CAR constructs, with CD3- and CD7-targeted nanoparticles achieving the highest levels of T-cell transfection *in vivo*, while reducing liver uptake and inducing measurable activation of circulating T cells. Functionally, these particles generated transient CD19 CAR expression in endogenous T cells, leading to significant B-cell depletion in blood and spleen, while cytokine levels remained dose-dependent and transient.

Box 2 **Lipid nanoparticles as enabling platforms for mRNA therapeutics**

Messenger RNA has emerged as a versatile therapeutic modality because it enables transient, programmable expression of virtually any protein without requiring genomic integration. However, the large size, negative charge, and susceptibility of naked mRNA to extracellular degradation severely limit its cellular uptake and intracellular availability. Lipid nanoparticles (LNPs) have become the dominant non-viral platform for overcoming these barriers and were central to the clinical translation of mRNA vaccines (123, 124). In addition to protecting mRNA from degradation, LNPs facilitate cellular uptake and promote release of the encapsulated cargo into the cytoplasm, where translation occurs. Their successful clinical implementation has established LNPs as a broadly applicable platform for mRNA vaccines, protein-replacement therapies, cancer immunotherapy, and genome engineering.

Conventional mRNA LNPs typically contain four principal components: an ionizable lipid, a phospholipid, cholesterol, and a PEG-conjugated lipid (125).The ionizable lipid is particularly important because it becomes positively charged under acidic conditions during nanoparticle formulation, enabling efficient association with negatively charged mRNA, while remaining largely neutral at physiological pH, thereby reducing nonspecific interactions and toxicity Following cellular uptake, interactions between ionizable lipids and endosomal membranes facilitate endosomal escape and cytoplasmic release of the mRNA; structure–activity studies of ionizable lipids have directly demonstrated that improved endosomal escape can substantially enhance mRNA delivery (126). The chemical structure and apparent pKa of the ionizable lipid, as well as the identity and relative proportions of the other lipid components, strongly influence particle size, stability, biodistribution, cellular uptake, endosomal escape, and ultimately mRNA. These parameters therefore provide multiple opportunities for engineering LNPs toward specific therapeutic applications.

These properties are particularly relevant to T-cell and CAR T-cell engineering. LNP-mediated delivery can provide transient CAR expression without the manufacturing complexity and genomic integration associated with viral vectors, while avoiding some of the viability losses associated with electroporation (51). Furthermore, the LNP surface can be functionalized with antibodies or other targeting ligands to promote selective interaction with T cells (52), and the lipid composition itself can be engineered to alter tissue and cell tropism. Such flexibility enables LNPs to function not simply as passive mRNA carriers, but as modular nanotechnological platforms in which cargo composition, delivery efficiency, targeting, and cellular stimulation can be independently optimized. These principles underlie both ex-vivo LNP-mediated CAR T-cell engineering and emerging strategies for direct *in vivo* generation of CAR T cells.

Despite their promise, several barriers remain before targeted LNP platforms can be broadly translated to *in vivo* CAR T-cell engineering. T-cell specificity remains incomplete, and LNP composition, particle size, surface targeting, and dose can strongly influence biodistribution, including uptake by the liver and other non-target cells, as well as innate immune activation. Early clinical studies demonstrate the feasibility of targeted mRNA-LNP-mediated CAR generation, but optimal dose, repeat-dosing schedules, durability, and long-term safety remain to be defined (127). Moreover, clinical translation will require stringent control of LNP critical quality attributes and batch-to-batch consistency, together with regulatory frameworks adapted to a therapy in which the engineered cellular product is generated directly within the patient (128).

Despite these promising advances and early clinical translation, several important questions remain regarding the safety, controllability, and therapeutic optimization of *in vivo* CAR T-cell generation approaches. Importantly, early clinical studies of targeted *in vivo* lentiviral CAR T-cell generation platforms, such as ESO-T01, have demonstrated the feasibility of generating functional CAR T cells directly within patients and have shown encouraging preliminary anti-tumor activity (148). However, significant toxicities, including cytokine release syndrome and immune effector cell-associated neurotoxicity syndrome, have also been reported. In a recently published clinical study of ESO-T01, five patients with relapsed or refractory multiple myeloma experienced grade 3 or higher adverse events following treatment (149). Reported toxicities included cytokine release syndrome, transient cytopenias, reversible elevations in hepatic enzymes, grade 2 infections, and grade 1 immune effector cell-associated neurotoxicity syndrome. Notably, one patient died during the study. Such results highlight the need for continued optimization of targeting specificity, biodistribution, safety-control mechanisms, and updated quality-control frameworks tailored to *in vivo* engineering strategies.

Another major unresolved challenge is the lack of an established optimal route for gene delivery. Systemic (intravenous, IV) and regional (lymph node–targeted) administration each offer distinct advantages and limitations, with important implications for therapeutic efficacy and treatment-associated toxicity. Among these, targeted delivery to lymph nodes represents a particularly attractive strategy for several reasons. Physiologically, T-cell priming naturally occurs within lymph nodes and is supported by the surrounding adipose tissue depots. Beyond containing high local densities of T cells, lymph nodes provide a highly supportive microenvironment enriched with homeostatic cytokines, including IL-7, IL-15, and IL-2, which promote T-cell activation, survival, proliferation, and differentiation. Furthermore, differentiating T cells continue to receive supportive cues as they leave the lymph node and enter circulation through the thoracic duct (150). The high concentration of resident T cells within lymph nodes also creates a localized target pool for gene delivery while reducing off-target transduction and collateral gene delivery that may accompany systemic administration.

Nanomaterial-specific safety must also be considered independently of the engineered T-cell product. Potential concerns include innate or adaptive immune responses to carrier components, complement activation, persistence or accumulation of non-degradable materials, toxicity of degradation products, and batch-dependent changes in biodistribution. These considerations become particularly important for *in vivo* engineering, where the final cellular product is generated within the patient and cannot be fully characterized before treatment; off-target gene delivery, dose control, biodistribution, and longitudinal monitoring therefore become central safety and regulatory considerations.

Collectively, these studies highlight the transformative potential of *in vivo* CAR T-cell engineering, and underscore the substantial biological, engineering, safety and controllability challenges that must be addressed before these approaches can become broadly scalable and clinically routine alternatives to conventional ex vivo manufacturing.

## 6 Nanotechnology for CAR T-cell manufacturing analytics and quality control

As CAR T-cell manufacturing evolves toward shorter, decentralized, and potentially automated production workflows, there is increasing need for rapid, integrated, and real-time analytical platforms capable of monitoring product quality throughout manufacturing. Current quality control (QC) strategies for CAR T-cell therapies remain labor-intensive, time-consuming, and highly dependent on centralized laboratory infrastructure. Standard release testing commonly includes sterility assays, viability measurements, immunophenotyping, vector copy number analysis, potency assays, and detection of replication-competent virus. While these approaches are essential for ensuring safety and consistency, they substantially contribute to manufacturing complexity and prolonged vein-to-vein times. In particular, sterility testing and product release remain major bottlenecks for rapid manufacturing approaches and fresh infusion workflows.

Nanotechnology-enabled sensing platforms offer promising solutions for next-generation CAR T-cell manufacturing analytics. Nanoscale biosensors possess high surface-to-volume ratios and tunable surface chemistries that enable highly sensitive detection of cellular, molecular, and metabolic biomarkers in small sample volumes and in real time. Such systems may enable continuous monitoring of CAR T-cell activation state, metabolic fitness, cytokine secretion, transduction efficiency, exhaustion marker expression, and contaminating microorganisms during manufacturing. Importantly, these technologies could facilitate closed-loop manufacturing systems in which analytical feedback dynamically informs culture conditions and process optimization.

Recent advances in nanomaterial-based biosensors and microfluidic analytical systems have enabled increasingly sensitive and multiplexed detection platforms relevant to cell therapy manufacturing. Electrochemical nanosensors, plasmonic nanoparticles, nanowire-based detectors, and impedance-based sensing systems have all demonstrated the ability to detect low-abundance biomolecules with rapid turnaround times. In the context of CAR T-cell manufacturing, such technologies may support rapid sterility testing, real-time monitoring of cytokine production, assessment of metabolic state, and detection of phenotypic shifts associated with differentiation or exhaustion. For example, microfluidic systems integrated with nanoscale sensing elements have been explored for single-cell profiling, extracellular vesicle analysis, and dynamic monitoring of secreted factors within small-volume cultures.

In addition to monitoring T-cell biology, nanotechnology-based QC systems may play an important role in decentralized and point-of-care manufacturing models. Miniaturized biosensors and integrated analytical cartridges could reduce dependence on centralized laboratories while enabling rapid product characterization directly within closed manufacturing systems. Such approaches may become particularly important for accelerated manufacturing workflows that aim to reduce production time from weeks to days or even hours. Moreover, integration of sensing technologies with automated manufacturing platforms may facilitate adaptive manufacturing strategies in which culture conditions, cytokine supplementation, or stimulation intensity are dynamically adjusted in response to real-time cellular readouts.

Notably, a related development in mRNA-based immunotherapy is the increasing use of artificial intelligence (AI) and machine-learning approaches to optimize multiple components of mRNA vaccines for efficient induction of antigen-specific T-cell responses. Deep-learning algorithms were shown to improve the identification and prioritization of immunogenic antigens and neoantigens by predicting peptide–MHC presentation and T-cell immunogenicity (151), while computational approaches are increasingly being used to optimize mRNA sequence features, including untranslated regions, to enhance antigen expression (152). AI has also been integrated with high-throughput screening to accelerate the discovery of ionizable lipids and optimize lipid nanoparticle formulations for mRNA delivery (153). Although these approaches have primarily been developed for mRNA vaccines rather than direct CAR T-cell generation, they illustrate how computational design can be combined with nanoscale delivery systems to optimize mRNA cargo, delivery efficiency, and ultimately T-cell stimulation. Such principles are closely related to the targeted mRNA-delivery strategies currently being explored for *in vivo* CAR T-cell engineering.

Despite these advances, significant translational challenges remain before nanotechnology-enabled QC platforms can be broadly implemented clinically. These include scalability, assay standardization, regulatory validation, integration with good manufacturing practice (GMP) workflows, and demonstration of reproducibility across heterogeneous patient-derived products. Nevertheless, the convergence of nanotechnology, biosensing, microfluidics, and cell manufacturing engineering has the potential to fundamentally reshape CAR T-cell production by enabling faster, more automated, and data-driven manufacturing systems with improved consistency and accessibility.

### 6.1 Regulatory and manufacturing considerations

Clinical translation of nanotechnology-enabled CAR T-cell manufacturing will require integration of nanomaterial-specific chemistry, manufacturing, and controls with established cell- and gene-therapy GMP requirements. A Quality-by-Design framework is particularly relevant because critical material attributes and process parameters—including particle size and distribution, composition, cargo encapsulation, ligand density, stability, and residual impurities—can directly influence potency, biodistribution, and safety. These attributes should therefore be linked to predefined critical quality attributes and controlled through validated in-process and release assays, with comparability established following scale-up or manufacturing changes. Regulatory evaluation will consequently extend beyond the final CAR T-cell phenotype to include reproducible characterization and manufacture of the nanomaterial or delivery component itself (128).

## 7 Summary, future directions, and emerging perspectives

This review summarizes how nanotechnology and nanomaterials are being integrated into the major stages of CAR T-cell manufacturing, including T-cell activation, genetic engineering, expansion, *in vivo* generation, and manufacturing analytics. Across these stages, engineered materials provide control over biochemical, spatial, and mechanical cues that influence not only conventional manufacturing metrics such as activation level, transduction efficiency, and cell yield, but also critical biological properties of the final CAR T-cell product, including differentiation, exhaustion, memory phenotype, metabolic fitness, persistence, and antitumor activity. In the activation stage, nanostructured and mechanically tunable platforms can regulate stimulation through properties such as topography, ligand organization, stiffness, and viscoelasticity, enabling more controlled T-cell priming than conventional rigid bead-based systems.

Nanotechnology also provides diverse strategies for CAR gene delivery and subsequent cell expansion. Nanoporous substrates and bioorthogonal targeting approaches can enhance viral transduction, whereas lipid nanoparticles and nanotube- or nanostraw-based systems enable non-viral delivery of CAR-encoding nucleic acids, in some cases combining activation and genetic engineering within a single platform. Engineered three-dimensional culture environments based on PEG–heparin, self-assembling peptides, collagen, and fibrin further demonstrate that the expansion microenvironment can be used to regulate both CAR T-cell proliferation and phenotype, including enrichment of memory-like and stem-like populations. Collectively, the studies reviewed here show that nanoscale material properties can function as active manufacturing parameters that shape both the efficiency of CAR T-cell production and the therapeutic characteristics of the resulting cells.

As CAR T-cell manufacturing evolves toward shorter, decentralized, and potentially automated production workflows, there is increasing need for rapid, integrated, and real-time analytical platforms capable of monitoring product quality throughout manufacturing. Current quality control (QC) strategies for CAR T-cell therapies remain labor-intensive, time-consuming, and highly dependent on centralized laboratory infrastructure. Standard release testing commonly includes sterility assays, viability measurements, immunophenotyping, vector copy number analysis, potency assays, and detection of replication-competent virus. While these approaches are essential for ensuring safety and consistency, they substantially contribute to manufacturing complexity and prolonged vein-to-vein times. In particular, sterility testing and product release remain major bottlenecks for rapid manufacturing approaches and fresh infusion workflows.

Nanotechnology-enabled sensing platforms offer promising solutions for next-generation CAR T-cell manufacturing analytics. Nanoscale biosensors possess high surface-to-volume ratios and tunable surface chemistries that enable highly sensitive detection of cellular, molecular, and metabolic biomarkers in small sample volumes and in real time (154–156). Such systems may enable continuous monitoring of CAR T-cell activation state, metabolic fitness, cytokine secretion, transduction efficiency, exhaustion marker expression, and contaminating microorganisms during manufacturing. Importantly, these technologies could facilitate closed-loop manufacturing systems in which analytical feedback dynamically informs culture conditions and process optimization.

Recent advances in nanomaterial-based biosensors and microfluidic analytical systems have enabled increasingly sensitive and multiplexed detection platforms relevant to cell therapy manufacturing. Electrochemical nanosensors (157, 158), plasmonic nanoparticles (159–161), nanowire-based detectors (162–164), and impedance-based sensing systems (165, 166) have all demonstrated the ability to detect low-abundance biomolecules with rapid turnaround times. In the context of CAR T-cell manufacturing, such technologies may support rapid sterility testing, real-time monitoring of cytokine production, assessment of metabolic state, and detection of phenotypic shifts associated with differentiation or exhaustion. In particular, impedance-based sensing systems have emerged as promising tools for real-time and label-free monitoring of CAR T-cell function during manufacturing and potency assessment (167, 168). These platforms enable continuous measurement of cell proliferation, activation dynamics, and immune cell-mediated cytotoxicity without requiring destructive endpoint assays, thereby providing a scalable approach for dynamic functional quality control. Such technologies may become increasingly valuable for automated and decentralized manufacturing workflows in which rapid assessment of CAR T-cell functionality is essential for accelerated product release and fresh infusion strategies. For example, microfluidic systems integrated with nanoscale sensing elements have been explored for single-cell profiling, extracellular vesicle analysis, and dynamic monitoring of secreted factors within small-volume cultures (169, 170).

In addition to monitoring T-cell biology, nanotechnology-based QC systems may play an important role in decentralized and point-of-care manufacturing models. Miniaturized biosensors and integrated analytical cartridges could reduce dependence on centralized laboratories while enabling rapid product characterization directly within closed manufacturing systems. Such approaches may become particularly important for accelerated manufacturing workflows that aim to reduce production time from weeks to days or even hours. Moreover, integration of sensing technologies with automated manufacturing platforms may facilitate adaptive manufacturing strategies in which culture conditions, cytokine supplementation, or stimulation intensity are dynamically adjusted in response to real-time cellular readouts.

Despite these advances, significant translational challenges remain before nanotechnology-enabled QC platforms can be broadly implemented clinically. These include scalability, assay standardization, regulatory validation, integration with good manufacturing practice (GMP) workflows, and demonstration of reproducibility across heterogeneous patient-derived products. Nevertheless, the convergence of nanotechnology, biosensing, microfluidics, and cell manufacturing engineering has the potential to fundamentally reshape CAR T-cell production by enabling faster, more automated, and data-driven manufacturing systems with improved consistency and accessibility.

The field of CAR T-cell manufacturing is undergoing a broader conceptual transition from static, labor-intensive production workflows toward dynamically engineered and biologically informed manufacturing systems. Historically, manufacturing strategies largely prioritized maximal expansion, high transduction efficiency, and standardized processing conditions. However, increasing evidence suggests that excessive ex-vivo manipulation can compromise T-cell fitness, persistence, and long-term therapeutic function. As a result, future manufacturing paradigms will likely place greater emphasis on preserving therapeutically favorable cellular states while minimizing ex vivo stress, processing time, and manufacturing complexity.

Nanotechnology and biomaterials engineering are uniquely positioned to accelerate this transition by enabling precise control over the physical, biochemical, and mechanical cues that regulate T-cell behavior. Rather than functioning solely as passive delivery materials, next-generation nanoscale platforms may actively program T-cell fate by modulating activation strength, differentiation trajectories, metabolic state, and exhaustion susceptibility. Emerging biomimetic systems capable of recreating aspects of the lymphoid niche, immune synapse architecture, or tissue mechanical environment may allow increasingly sophisticated control over CAR T-cell phenotype and function during manufacturing (171–173).

At the same time, the boundaries between ex vivo and *in vivo* manufacturing approaches are beginning to converge. Advances in targeted nanoparticles, engineered viral vectors, and programmable delivery systems raise the possibility of generating CAR T cells directly within patients, potentially bypassing many of the logistical and economic barriers associated with centralized manufacturing. While substantial safety and regulatory challenges remain, *in vivo* engineering strategies may ultimately expand accessibility and enable more rapid treatment for patients with aggressive disease.

The economic value of nanotechnology-enabled manufacturing will ultimately depend on whether reductions in processing time, labor, facility requirements, and product variability offset the added costs of specialized materials, fabrication, and analytical control. Platforms that combine manufacturing steps or reduce ex vivo culture may lower overall manufacturing burden, whereas complex nanofabrication or bespoke nanoparticle production may shift costs upstream. Commercial feasibility will therefore depend on scalable GMP production and demonstrable reductions in total manufacturing and treatment costs rather than material performance alone (174).

Another major future direction involves the integration of sensing technologies, automation, and computational analysis into CAR T-cell manufacturing pipelines. Real-time biosensing platforms, microfluidic monitoring systems, and AI-guided process optimization may enable adaptive manufacturing workflows in which culture conditions are continuously adjusted based on dynamic cellular readouts. Such systems could reduce product variability, improve reproducibility across patients, and support decentralized or point-of-care manufacturing models.

Importantly, future advances in CAR T-cell manufacturing will likely require balancing multiple competing biological objectives, including activation strength, transduction efficiency, proliferative capacity, persistence, exhaustion resistance, and safety. In this context, nanoscale materials offer powerful opportunities to precisely tune these parameters in ways that are difficult to achieve using conventional manufacturing approaches. Continued collaboration between immunologists, materials scientists, bioengineers, clinicians, and manufacturing specialists will therefore be essential to translate these technologies into clinically scalable platforms.

Collectively, emerging nanotechnology-enabled approaches are redefining CAR T-cell manufacturing from a largely empirical production process into a programmable engineering discipline. By integrating advances in biomaterials, mechanobiology, gene delivery, biosensing, and automation, next-generation manufacturing strategies may improve not only the scalability, and accessibility of CAR T-cell therapies, but also the biological quality and therapeutic duration of he final cellular product. Continued convergence between immunology, nanotechnology, and bioengineering will likely play a central role in shaping future cellular immunotherapies capable of broader clinical translation across both hematologic and solid malignancies.

## References

[1] Grupp SA Kalos M Barrett D Aplenc R Porter DL Rheingold SR . Chimeric antigen receptor-modified T cells for acute lymphoid leukemia. N Engl J Med. (2013) 368:1509–18. doi: 10.1056/NEJMoa1215134 23527958 PMC4058440

[2] Eshhar Z Waks T Gross G Schindler DG . Specific activation and targeting of cytotoxic lymphocytes through chimeric single chains consisting of antibody-binding domains and the y or C subunits of the immunoglobulin and T-cell receptors. Proc Natl Acad Sci USA. (1993) 90:720–4. doi: 10.1073/pnas.90.2.720 8421711 PMC45737

[3] Milone MC Fish JD Carpenito C Carroll RG Binder GK Teachey D . Chimeric receptors containing CD137 signal transduction domains mediate enhanced survival of T cells and increased antileukemic efficacy *in vivo*. Mol Ther J Am Soc Gene Ther. (2009) 17:1453–64. doi: 10.1038/mt.2009.83 19384291 PMC2805264

[4] Sterner RC Sterner RM . CAR-T cell therapy: Current limitations and potential strategies. Blood Cancer J. (2021) 11:69. doi: 10.1038/s41408-021-00459-7 33824268 PMC8024391

[5] Gómez-Melero S Hassouneh F Vallejo-Bermúdez IM Agüera-Morales E Solana R Caballero-Villarraso J . Tandem CAR-T cell therapy: Recent advances and current challenges. Front Immunol. (2025) 16:1546172. doi: 10.3389/fimmu.2025.1546172 40092990 PMC11907001

[6] Jan N Shah H Khan S Khan NU Li Y Zhang X . T cell-inspired therapeutic delivery platforms: From nanomedicines to cell therapy. Mater Today Bio. (2026) 37:102909. doi: 10.1016/j.mtbio.2026.102909 41732384 PMC12925206

[7] Rafei H Upadhyay R Sharma P . A guide to CAR T cell therapies: Development, current status and future prospects. Nat Rev Immunol. (2026). doi: 10.1038/s41577-026-01322-1 42393377

[8] Cappell KM Kochenderfer JN . Long-term outcomes following CAR T cell therapy: What we know so far. Nat Rev Clin Oncol. (2023) 20:359–71. doi: 10.1038/s41571-023-00754-1 37055515 PMC10100620

[9] Srivastava R . Stimuli-responsive nanomaterials for the precision delivery of mRNA cancer vaccines. Nano Trends. (2025) 11:100147. doi: 10.1016/j.nwnano.2025.10014742574925

[10] Srivastava R . AI-powered mapping of tumor immunity for optimized mRNA vaccine engineering. Front Oncol. (2026) 16:1766201. doi: 10.3389/fonc.2026.1766201 41853314 PMC12992067

[11] Fraietta JA Lacey SF Orlando EJ Pruteanu-Malinici I Gohil M Lundh S . Determinants of response and resistance to CD19 chimeric antigen receptor (CAR) T cell therapy of chronic lymphocytic leukemia. Nat Med. (2018) 24:563–71. doi: 10.1038/s41591-018-0010-1 29713085 PMC6117613

[12] Ayala Ceja M Khericha M Harris CM Puig-Saus C Chen YY . CAR-T cell manufacturing: Major process parameters and next-generation strategies. J Exp Med. (2024) 221:e20230903. doi: 10.1084/jem.20230903 38226974 PMC10791545

[13] Gauthier J Bezerra ED Hirayama AV Fiorenza S Sheih A Chou CK . Factors associated with outcomes after a second CD19-targeted CAR T-cell infusion for refractory B-cell malignancies. Blood. (2021) 137:323–35. doi: 10.1182/blood.2020006770 32967009 PMC7819764

[14] Klebanoff CA Scott CD Leonardi AJ Yamamoto TN Cruz AC Ouyang C . Memory T cell-driven differentiation of naive cells impairs adoptive immunotherapy. J Clin Invest. (2016) 126:318–34. doi: 10.1172/JCI81217 26657860 PMC4701537

[15] Gattinoni L Lugli E Ji Y Pos Z Paulos CM Quigley MF . A human memory T cell subset with stem cell-like properties. Nat Med. (2011) 17:1290–7. doi: 10.1038/nm.2446 21926977 PMC3192229

[16] Ghassemi S Nunez-Cruz S O’Connor RS Fraietta JA Patel PR Scholler J . Reducing ex vivo culture improves the antileukemic activity of chimeric antigen receptor (CAR) T cells. Cancer Immunol Res. (2018) 6:1100–9. doi: 10.1158/2326-6066.CIR-17-0405 30030295 PMC8274631

[17] Long AH Haso WM Shern JF Wanhainen KM Murgai M Ingaramo M . 4-1BB costimulation ameliorates T cell exhaustion induced by tonic signaling of chimeric antigen receptors. Nat Med. (2015) 21:581–90. doi: 10.1038/nm.3838 25939063 PMC4458184

[18] Philip M Fairchild L Sun L Horste EL Camara S Shakiba M . Chromatin states define tumour-specific T cell dysfunction and reprogramming. Nature. (2017) 545:452–6. doi: 10.1038/nature22367 28514453 PMC5693219

[19] Balke-Want H Keerthi V Cadinanos-Garai A Fowler C Gkitsas N Brown AK . Non-viral chimeric antigen receptor (CAR) T cells going viral. Immuno-Oncol Technol. (2023) 18:100375. doi: 10.1016/j.iotech.2023.100375 37124148 PMC10139995

[20] Moretti A Ponzo M Nicolette CA Tcherepanova IY Biondi A Magnani CF . The past, present, and future of non-viral CAR T cells. Front Immunol. (2022) 13:867013. doi: 10.3389/fimmu.2022.867013 35757746 PMC9218214

[21] Lukjanov V Koutná I Šimara P . CAR T-cell production using nonviral approaches. J Immunol Res. (2021) 2021:6644685. doi: 10.1155/2021/6644685 33855089 PMC8019376

[22] Rossi M Breman E . Engineering strategies to safely drive CAR T-cells into the future. Front Immunol. (2024) 15:1411393. doi: 10.3389/fimmu.2024.1411393 38962002 PMC11219585

[23] Tao Z Chyra Z Kotulová J Celichowski P Mihályová J Charvátová S . Impact of T cell characteristics on CAR-T cell therapy in hematological malignancies. Blood Cancer J. (2024) 14:213. doi: 10.1038/s41408-024-01193-6 39627220 PMC11615218

[24] Peng J-J Wang L Li Z Ku C-L Ho P-C . Metabolic challenges and interventions in CAR T cell therapy. Sci Immunol. (2023) 8:eabq3016. doi: 10.1126/sciimmunol.abq3016 37058548

[25] Bulliard Y Andersson BS Baysal MA Damiano J Tsimberidou AM . Reprogramming T cell differentiation and exhaustion in CAR-T cell therapy. J Hematol OncolJ Hematol Oncol. (2023) 16:108. doi: 10.1186/s13045-023-01504-7 37880715 PMC10601191

[26] Diorio C Teachey DT Grupp SA . Allogeneic chimeric antigen receptor cell therapies for cancer: Progress made and remaining roadblocks. Nat Rev Clin Oncol. (2025) 22:10–27. doi: 10.1038/s41571-024-00959-y 39548270

[27] Bot A Scharenberg A Friedman K Guey L Hofmeister R Andorko JI . *In vivo* chimeric antigen receptor (CAR)-T cell therapy. Nat Rev Drug Discov. (2026) 25:116–37. doi: 10.1038/s41573-025-01291-5 41028170

[28] Liu Y Crawford BM Vo-Dinh T . Gold nanoparticles-mediated photothermal therapy and *immunotherapy*. Immunotherapy. (2018) 10:1175–88. doi: 10.2217/imt-2018-0029 30236026

[29] Mitchell MJ Billingsley MM Haley RM Wechsler ME Peppas NA Langer R . Engineering precision nanoparticles for drug delivery. Nat Rev Drug Discov. (2021) 20:101–24. doi: 10.1038/s41573-020-0090-8 33277608 PMC7717100

[30] Siriwon N Kim YJ Siegler E Chen X Rohrs JA Liu Y . CAR-T cells surface-engineered with drug-encapsulated nanoparticles can ameliorate intratumoral T-cell hypofunction. Cancer Immunol Res. (2018) 6:812–24. doi: 10.1158/2326-6066.CIR-17-0502 29720380

[31] Tang L Zheng Y Melo MB Mabardi L Castaño AP Xie Y-Q . Enhancing T cell therapy through TCR-signaling-responsive nanoparticle drug delivery. Nat Biotechnol. (2018) 36:707–16. doi: 10.1038/nbt.4181 29985479 PMC6078803

[32] Stephan MT Stephan SB Bak P Chen J Irvine DJ . Synapse-directed delivery of immunomodulators using T-cell-conjugated nanoparticles. Biomaterials. (2012) 33:5776–87. doi: 10.1016/j.biomaterials.2012.04.029 22594972 PMC3395588

[33] Kiru L Zlitni A Tousley AM Dalton GN Wu W Lafortune F . *In vivo* imaging of nanoparticle-labeled CAR T cells. Proc Natl Acad Sci. (2022) 119:e2102363119. doi: 10.1073/pnas.2102363119 35101971 PMC8832996

[34] Zhang F Stephan SB Ene CI Smith TT Holland EC Stephan MT . Nanoparticles that reshape the tumor milieu create a therapeutic window for effective T-cell therapy in solid malignancies. Cancer Res. (2018) 78:3718–30. doi: 10.1158/0008-5472.CAN-18-0306 29760047 PMC6030470

[35] Chen H Machado A An D Becharef S Autret G Ayollo D . *In vivo* monitoring and magnetically-enhanced delivery of CAR T-cells to solid tumor. Adv Funct Mater. (2025) 35:2414368. doi: 10.1002/adfm.20241436842587375

[36] Xie T Chen X Fang J Xue W Zhang J Tong H . Non-invasive monitoring of the kinetic infiltration and therapeutic efficacy of nanoparticle-labeled chimeric antigen receptor T cells in glioblastoma via 7.0-tesla magnetic resonance imaging. Cytotherapy. (2021) 23:211–22. doi: 10.1016/j.jcyt.2020.10.006 33334686

[37] Dubois VP Sehl OC Foster PJ Ronald JA . Visualizing CAR-T cell immunotherapy using 3 Tesla fluorine-19 MRI. Mol Imaging Biol. (2022) 24:298–308. doi: 10.1007/s11307-021-01672-3 34786668 PMC8983548

[38] Geng Y Wang Z Liu X Yao H Sun J Lao Y-H . Nano-therapeutics for *in vivo* immune cell engineering. Small Methods. (2026) 10:e01973. doi: 10.1002/smtd.202501973 42174402

[39] Gong N Sheppard NC Billingsley MM June CH Mitchell MJ . Nanomaterials for T-cell cancer immunotherapy. Nat Nanotechnol. (2021) 16:25–36. doi: 10.1038/s41565-020-00822-y 33437036

[40] Lafuente-Gómez N Kang S Mooney DJ . Nanotechnology for CAR T cells and tumour-infiltrating lymphocyte therapies. Nat Nanotechnol. (2025) 20:1186–98. doi: 10.1038/s41565-025-02008-w 40931058

[41] Long J Wang Y Jiang X Ge J Chen M Zheng B . Nanomaterials boost CAR-T therapy for solid tumors. Adv Healthc Mater. (2024) 13:e2304615. doi: 10.1002/adhm.202304615 38483400

[42] Casati A Varghaei-Nahvi A Feldman SA Assenmacher M Rosenberg SA Dudley ME . Clinical-scale selection and viral transduction of human naïve and central memory CD8+ T cells for adoptive cell therapy of cancer patients. Cancer Immunol Immunother CII. (2013) 62:1563–73. doi: 10.1007/s00262-013-1459-x 23903715 PMC6348480

[43] Cheung AS Zhang DKY Koshy ST Mooney DJ . Scaffolds that mimic antigen-presenting cells enable ex vivo expansion of primary T cells. Nat Publ Group. (2018) 36:160–9. doi: 10.1038/nbt.4047 29334370 PMC5801009

[44] Zhang DKY Adu-Berchie K Iyer S Liu Y Cieri N Brockman JM . Enhancing CAR-T cell functionality in a patient-specific manner. Nat Commun. (2023) 14:506. doi: 10.1038/s41467-023-36126-7 36720856 PMC9889707

[45] Yassin AA-K Ureña Martin C Le Saux G Pandey A Tzadka S Toledo E . Mechanostimulatory platform for improved CAR T cell immunotherapy. Adv Mat. (2025) 37:e2412482. doi: 10.1002/adma.202412482 40348587

[46] Tzdaka S Singh SSE Yassin AAK Toledo E Pandit JJ Porgador A . A high-quality and -throughput colloidal lithography by mechanical assembly and ice-based transfer. Nanoscale. (2025) 17:22134–44. doi: 10.1039/D5NR02468D 40960887

[47] Liu Z Li Y-R Yang Y Zhu Y Yuan W Hoffman T . Viscoelastic synthetic antigen-presenting cells for augmenting the potency of cancer therapies. Nat BioMed Eng. (2024) 8:1615–33. doi: 10.1038/s41551-024-01272-w 39455719 PMC12833746

[48] Aramesh M Yu D Essand M Persson C . Enhanced cellular uptake through nanotopography-induced macropinocytosis. Adv Funct Mater. (2024) 34:2400487. doi: 10.1002/adfm.20240048742587375

[49] Pan H Li P Li G Li W Hu B He H . Glycometabolic bioorthogonal chemistry-guided viral transduction for robust human T cell engineering. Adv Funct Mater. (2019) 29:1807528. doi: 10.1002/adfm.20180752842587375

[50] Billingsley MM Singh N Ravikumar P Zhang R June CH Mitchell MJ . Ionizable lipid nanoparticle-mediated mRNA delivery for human CAR T cell engineering. Nano Lett. (2020) 20:1578–89. doi: 10.1021/acs.nanolett.9b04246 31951421 PMC7313236

[51] Billingsley MM Hamilton AG Mai D Patel SK Swingle KL Sheppard NC . Orthogonal design of experiments for optimization of lipid nanoparticles for mRNA engineering of CAR T cells. Nano Lett. (2022) 22:533–42. doi: 10.1021/acs.nanolett.1c02503 34669421 PMC9335860

[52] Metzloff AE Padilla MS Gong N Billingsley MM Han X Merolle M . Antigen presenting cell mimetic lipid nanoparticles for rapid mRNA CAR T cell cancer immunotherapy. Adv Mater. (2024) 36:e2313226. doi: 10.1002/adma.202313226 38419362 PMC11209815

[53] Chen Y Mach M Shokouhi A-R Yoh HZ Bishop DC Murayama T . Efficient non-viral CAR-T cell generation via silicon-nanotube-mediated transfection. Mater Today. (2023) 63:8–17. doi: 10.1016/J.MATTOD.2023.02.00942574925

[54] Shokouhi A-R Chen Y Yoh HZ Brenker J Alan T Murayama T . Engineering efficient CAR-T cells via electroactive nanoinjection. Adv Mater. (2023) 35:2304122. doi: 10.1002/adma.202304122 37434421

[55] Castellote-Borrell M Domingo M Merlina F Lu H Colell S Bachiller M . Lymph-node inspired hydrogels enhance CAR expression and proliferation of CAR T cells. ACS Appl Mater Interfaces. (2025) 17:16548–60. doi: 10.1021/acsami.4c19942 40042178 PMC11931490

[56] Jie J Mao D Cao J Feng P Yang P . Customized multifunctional peptide hydrogel scaffolds for CAR-T-cell rapid proliferation and solid tumor immunotherapy. ACS Appl Mater Interfaces. (2022) 14:37514–27. doi: 10.1021/acsami.2c10727 35944246

[57] Adu-Berchie K Liu Y Zhang DKY Freedman BR Brockman JM Vining KH . Generation of functionally distinct T-cell populations by altering the viscoelasticity of their extracellular matrix. Nat BioMed Eng. (2023) 7:1374–91. doi: 10.1038/s41551-023-01052-y 37365267 PMC10749992

[58] Ghassemi S Durgin JS Nunez-Cruz S Patel J Leferovich J Pinzone M . Rapid manufacturing of non-activated potent CAR T cells. Nat BioMed Eng. (2022) 6:118–28. doi: 10.1038/S41551-021-00842-6 35190680 PMC8860360

[59] Valitutti S Miller S Cella M Padovan E Lanzavecchia A . Serial triggering of many T-cell receptors by a few peptide–MHC complexes. Nat 1995 3756527. (1995) 375:148–51. doi: 10.1038/375148a0 7753171

[60] Feng Y Reinherz EL Lang MJ . Aβ T cell receptor mechanosensing forces out serial engagement. Trends Immunol. (2018) 39:596–609. doi: 10.1016/j.it.2018.05.005 30060805 PMC6154790

[61] Hellmeier J Platzer R Eklund AS Schlichthaerle T Karner A Motsch V . DNA origami demonstrate the unique stimulatory power of single pMHCs as T cell antigens. Proc Natl Acad Sci. (2021) 118:e2016857118. doi: 10.1073/pnas.2016857118 33468643 PMC7848602

[62] Chen B-M Al-Aghbar MA Lee C-H Chang T-C Su Y-C Li Y-C . The affinity of elongated membrane-tethered ligands determines potency of T cell receptor triggering. Front Immunol. (2017) 8. doi: 10.3389/fimmu.2017.00793 28740495 PMC5502409

[63] Wells AD Gudmundsdottir H Turka LA . Following the fate of individual T cells throughout activation and clonal expansion. Signals from T cell receptor and CD28 differentially regulate the induction and duration of a proliferative response. J Clin Invest. (1997) 100:3173–83. doi: 10.1172/JCI119873 9399965 PMC508531

[64] Courtney AH Lo W-L Weiss A . TCR signaling: Mechanisms of initiation and propagation. Trends Biochem Sci. (2018) 43:108–23. doi: 10.1016/j.tibs.2017.11.008 29269020 PMC5801066

[65] Dustin ML . Recent advances in understanding TCR signaling: A synaptic perspective. Fac Rev. (2023) 12:25. doi: 10.12703/r/12-25 37900153 PMC10608137

[66] Davis SJ van der Merwe PA . The kinetic-segregation model: TCR triggering and beyond. Nat Immunol. (2006) 7:803–9. doi: 10.1038/ni1369 16855606

[67] Minguet S Swamy M Alarcón B Luescher IF Schamel WWA . Full activation of the T cell receptor requires both clustering and conformational changes at CD3. Immunity. (2007) 26:43–54. doi: 10.1016/j.immuni.2006.10.019 17188005

[68] Huse M . Mechanical forces in the immune system. Nat Rev Immunol. (2017) 17:679–90. doi: 10.1038/nri.2017.74 28757604 PMC6312705

[69] Basu R Whitlock BM Husson J Le Floc’h A Jin W Oyler-Yaniv A . Cytotoxic T *cell*s use mechanical force to potentiate target *cell* killing. Cell. (2016) 165:100–10. doi: 10.1016/j.cell.2016.01.021 26924577 PMC4808403

[70] O’Connor RS Hao X Shen K Bashour K Akimova T Hancock WW . Substrate rigidity regulates human T cell activation and proliferation. J Immunol Baltim Md 1950. (2012) 189:1330–9. doi: 10.4049/jimmunol.1102757 22732590 PMC3401283

[71] Saitakis M Dogniaux S Goudot C Bufi N Asnacios S Maurin M . Different TCR-induced T lymphocyte responses are potentiated by stiffness with variable sensitivitydifferent TCR-induced T lymphocyte responses are potentiated by stiffness with variable sensitivity. eLife. (2017) 6:1–29. doi: 10.7554/eLife.23190 28594327 PMC5464771

[72] Orbach R Su X . Surfing on membrane waves: Microvilli, curved membranes, and immune signaling. Front Immunol. (2020) 11:2187. doi: 10.3389/fimmu.2020.02187 33013920 PMC7516127

[73] Al-Aghbar MA Jainarayanan AK Dustin ML Roffler SR . The interplay between membrane topology and mechanical forces in regulating T cell receptor activity. Commun Biol 2022 51. (2022) 5:1–16. doi: 10.1038/s42003-021-02995-1 35017678 PMC8752658

[74] Cai E Marchuk K Beemiller P Beppler C Rubashkin MG Weaver VM . Visualizing dynamic microvillar search and stabilization during ligand detection by T cells. Science. (2017) 356:1–10. doi: 10.1126/science.aal3118 28495700 PMC6364556

[75] Jung Y Riven I Feigelson SW Kartvelishvily E Tohya K Miyasaka M . Three-dimensional localization of T-cell receptors in relation to microvilli using a combination of superresolution microscopies. Proc Natl Acad Sci. (2016) 113:E5916–24. doi: 10.1073/pnas.1605399113 27647916 PMC5056101

[76] Pérez-Ferreros P Gaus K Goyette J . Tethered signaling in inhibitory immune receptors. Front Phys. (2019) 6:158. doi: 10.3389/fphy.2018.00158

[77] Bhingardive V Kossover A Iraqi M Khand B Le Saux G Porgador A . Antibody-functionalized nanowires: A tuner for the activation of T cells. Nano Lett. (2021) 21:4241–8. doi: 10.1021/acs.nanolett.1c00245 33989498

[78] Aramesh M Stoycheva D Sandu I Ihle SJ Zünd T Shiu J-Y . Nanoconfinement of microvilli alters gene expression and boosts T cell activation. Proc Natl Acad Sci. (2021) 118:e2107535118. doi: 10.1073/pnas.2107535118 34599101 PMC8501847

[79] Pageon SV Tabarin T Yamamoto Y Ma Y Bridgeman JS Cohnen A . Functional role of T-cell receptor nanoclusters in signal initiation and antigen discrimination. Proc Natl Acad Sci. (2016) 113:E5454–63. doi: 10.1073/pnas.1607436113 27573839 PMC5027455

[80] Dustin ML Groves JT . Receptor signaling clusters in the immune synapse. Annu Rev Biophys. (2012) 41:543–56. doi: 10.1146/annurev-biophys-042910-155238 22404679 PMC4000727

[81] Dustin ML . What counts in the immunological synapse? Mol Cell. (2014) 54:255–62. doi: 10.1016/J.MOLCEL.2014.04.001 24766889 PMC4005017

[82] Matic J Deeg J Scheffold A Goldstein I Spatz JP . Fine tuning and efficient T cell activation with stimulatory aCD3 nanoarrays. Nano Lett. (2013) 13:5090–7. doi: 10.1021/nl4022623 24111628 PMC3834297

[83] Deeg J Axmann M Matic J Liapis A Depoil D Afrose J . T cell activation is determined by the number of presented antigens. Nano Lett. (2013) 13:5619–26. doi: 10.1021/nl403266t 24117051 PMC3828117

[84] Cai H Muller J Depoil D Mayya V Sheetz MP Dustin ML . Full control of ligand positioning reveals spatial thresholds for T cell receptor triggering. Nat Nanotechnol. (2018) 13:610–7. doi: 10.1038/s41565-018-0113-3 29713075 PMC6035778

[85] Delcassian D Depoil D Rudnicka D Liu M Davis DM Dustin ML . Nanoscale ligand spacing influences receptor triggering in T cells and NK cells. Nano Lett. (2013) 13:5608–14. doi: 10.1021/nl403252x 24125583 PMC4288448

[86] Le Saux G Nowakowski P Toledo E Tzdaka S Naaz S Segal Y . Tiny clusters for big impact in T cell activation. Nano Lett. (2025) 25:11878–87. doi: 10.1021/acs.nanolett.5c02361 40765318

[87] Jenkins E Whitehead T Fellermeyer M Davis SJ Sharma S . The current state and future of T-cell exhaustion research. Oxf Open Immunol. (2023) 4:iqad006. doi: 10.1093/oxfimm/iqad006 37554723 PMC10352049

[88] Hinrichs CS Borman ZA Gattinoni L Yu Z Burns WR Huang J . Human effector CD8+ T cells derived from naive rather than memory subsets possess superior traits for adoptive immunotherapy. Blood. (2011) 117:808–14. doi: 10.1182/blood-2010-05-286286 20971955 PMC3035075

[89] Graef P Buchholz VR Stemberger C Flossdorf M Henkel L Schiemann M . Serial transfer of single-cell-derived immunocompetence reveals stemness of CD8(+) central memory T cells. Immunity. (2014) 41:116–26. doi: 10.1016/j.immuni.2014.05.018 25035956

[90] Judokusumo E Tabdanov E Kumari S Dustin ML Kam LC . Mechanosensing in T lymphocyte activation. Biophys J. (2012) 102:L5–7. doi: 10.1016/j.bpj.2011.12.011 22339876 PMC3260692

[91] Lambert LH Goebrecht GKE De Leo SE O’Connor RS Nunez-Cruz S Li TD . Improving T cell expansion with a soft touch. Nano Lett. (2017) 17:821–6. doi: 10.1021/acs.nanolett.6b04071 28122453 PMC5504474

[92] Dang AP De Leo S Bogdanowicz DR Yuan DJ Fernandes SM Brown JR . Enhanced activation and expansion of T cells using mechanically soft elastomer fibers. Adv Biosyst. (2018) 2:1700167. doi: 10.1002/adbi.201700167 31008184 PMC6469863

[93] Anandasivam N Ali R Gustinvil L Rosenwasser MJ Dunlop IE Delcassian D . Tunable enhancement of T cell expansion through modulation of stiffness and adhesion receptor engagement in an engineered hydrogel platform. Adv Mater. (2026) 38:e05965. doi: 10.1002/adma.202505965 41504553 PMC12921338

[94] du Roure O Saez A Buguin A Austin RH Chavrier P Silberzan P . Force mapping in epithelial cell migration. Proc Nat Acad Sci USA. (2005) 102:2390–5. doi: 10.1073/pnas.0408482102 15695588 PMC548966

[95] Balaban NQ Schwarz US Riveline D Goichberg P Tzur G Sabanay I . Force and focal adhesion assembly: A close relationship studied using elastic micropatterned substrates. Nat Cell Biol. (2001) 3:466–72. doi: 10.1038/35074532 11331874

[96] Ghassemi S Meacci G Liu S Gondarenko AA Mathur A Roca-Cusachs P . Cells test substrate rigidity by local contractions on submicrometer pillars. Proc Natl Acad Sci USA. (2012) 109:5328–33. doi: 10.1073/pnas.1119886109 22431603 PMC3325713

[97] Jin W Tamzalit F Chaudhuri PK Black CT Huse M Kam LC . T cell activation and immune synapse organization respond to the microscale mechanics of structured surfaces. Proc Natl Acad Sci. (2019) 116:19835–40. doi: 10.1073/pnas.1906986116 31527238 PMC6778209

[98] Pandey A Iraqi M Toledo E Al-Kader Yassin A Podvalni E Naaz S . Elastic microstructures: Combining biochemical, mechanical, and topographical cues for the effective activation and proliferation of cytotoxic T cells. ACS Appl Mater Interfaces. (2023) 15:31103–13. doi: 10.1021/acsami.3c01871 37347217

[99] Chaudhuri PK Wang MS Black CT Huse M Kam LC . Modulating T cell activation using depth sensing topographic cues. Adv Biosyst. (2020) 4:2000143. doi: 10.1002/adbi.202000143 32744809 PMC8404344

[100] Prinz CN . Interactions between semiconductor nanowires and living cells. J Phys Condens Matter. (2015) 27:233103–14. doi: 10.1088/0953-8984/27/23/233103 26010455

[101] Le Saux G Bar-Hanin N Edri A Hadad U Porgador A Schvartzman M . Nanoscale mechanosensing of natural killer cells is revealed by antigen-functionalized nanowires. Adv Mater. (2019) 31:1805954. doi: 10.1002/adma.201805954 30488552

[102] Bhingardive V Le Saux G Edri A Porgador A Schvartzman M . Nanowire based guidance of the morphology and cytotoxic activity of natural killer cells. Small. (2021) 17:2007347. doi: 10.1002/smll.202007347 33719212

[103] Bhingardive V Edri A Kossover A Saux GL Khand B Radinsky O . Nanowire based mechanostimulating platform for tunable activation of natural killer cells. Adv Funct Mater. (2021) 31:2103063. doi: 10.1002/ADFM.20210306342587375

[104] Van Der Merwe PA Dushek O . Mechanisms for T cell receptor triggering. Nat Rev Immunol. (2011) 11:47–55. doi: 10.1038/nri2887 21127503

[105] Choudhuri K Wiseman D Brown MH Gould K Van Der Merwe PA . T-cell receptor triggering is critically dependent on the dimensions of its peptide-MHC ligand. Nature. (2005) 436:578–82. doi: 10.1038/nature03843 16049493

[106] Esmaeili F Wu YL Wang Z Abdrabou A Juska VB Zargartalebi H . Spiky gold nanoparticles, a nanoscale approach to enhanced ex vivo T-cell activation. ACS Nano. (2024) 18:21554–64. doi: 10.1021/acsnano.4c07306 39079006

[107] Goyette J Nieves DJ Ma Y Gaus K . How does T cell receptor clustering impact on signal transduction? J Cell Sci. (2019) 132:jcs226423. doi: 10.1242/jcs.226423 30745330

[108] Doh J Irvine DJ . Immunological synapse arrays: Patterned protein surfaces that modulate immunological synapse structure formation in T cells. Proc Nat Acad Sci USA. (2006) 103:5700–5. doi: 10.1073/pnas.0509404103 16585528 PMC1458636

[109] Arnold M Cavalcanti-Adam EA Glass R Blümmel J Eck W Kantlehner M . Activation of integrin function by nanopatterned adhesive interfaces. ChemPhysChem. (2004) 5:383–8. doi: 10.1002/cphc.200301014 15067875

[110] Schvartzman M Palma M Sable J Abramson J Hu X Sheetz MP . Nanolithographic control of the spatial organization of cellular adhesion receptors at the single-molecule level. Nano Lett. (2011) 11:1306–12. doi: 10.1021/nl104378f 21319842 PMC3061283

[111] Cai H Wolfenson H Depoil D Dustin ML Sheetz MP Wind SJ . Molecular occupancy of nanodot arrays. ACS Nano. (2016) 10:4173–83. doi: 10.1021/acsnano.5b07425 26966946 PMC5337305

[112] Keydar Y Le Saux G Pandey A Avishay E Bar-Hanin N Esti T . Natural killer cells’ immune response requires a minimal *nanoscale* distribution of activating antigens. Nanoscale. (2018) 10:14651–9. doi: 10.1039/C8NR04038A 30033475

[113] Pandey A Nowakowski P Ureña Martin C Abu Ahmad M Edri A Toledo E . Membrane fluctuation model for understanding the effect of receptor nanoclustering on the activation of natural killer cells through biomechanical feedback. Nano Lett. (2024) 24:5395–402. doi: 10.1021/acs.nanolett.3c02815 38684070

[114] Chaudhuri O Cooper-White J Janmey PA Mooney DJ Shenoy VB . Effects of extracellular matrix viscoelasticity on cellular behaviour. Nature. (2020) 584:535–46. doi: 10.1038/s41586-020-2612-2 32848221 PMC7676152

[115] Dan L Kang-Zheng L . Optimizing viral transduction in immune cell therapy manufacturing: Key process design considerations. J Transl Med. (2025) 23:501. doi: 10.1186/s12967-025-06524-0 40316943 PMC12046913

[116] de la Mota S Suchet L van Wijk M Stibbs DJ Silva Couto P Rafiq QA . On the road to *in vivo* CAR-T success: Comparing promising viral and non-viral vectors. Biotechnol Adv. (2026) 90:108907. doi: 10.1016/j.biotechadv.2026.108907 42049130

[117] Hu Q Zhao H Zhou K Tian X Wang Q Hua X . Scarless circular mRNA-based CAR-T cell therapy elicits superior antitumor efficacy. Signal Transduct Target Ther. (2025) 10:411. doi: 10.1038/s41392-025-02512-4 41430032 PMC12722250

[118] Beatty GL Haas AR Maus MV Torigian DA Soulen MC Plesa G . Mesothelin-specific chimeric antigen receptor mRNA-engineered T cells induce anti-tumor activity in solid malignancies. Cancer Immunol Res. (2014) 2:112–20. doi: 10.1158/2326-6066.CIR-13-0170 24579088 PMC3932715

[119] Zhao Y Moon E Carpenito C Paulos CM Liu X Brennan AL . Multiple injections of electroporated autologous T cells expressing a chimeric antigen receptor mediate regression of human disseminated tumor. Cancer Res. (2010) 70:9053–61. doi: 10.1158/0008-5472.CAN-10-2880 20926399 PMC2982929

[120] Locke FL Ghobadi A Jacobson CA Miklos DB Lekakis LJ Oluwole OO . Long-term safety and activity of axicabtagene ciloleucel in refractory large B-cell lymphoma (ZUMA-1): A single-arm, multicentre, phase 1-2 trial. Lancet Oncol. (2019) 20:31–42. doi: 10.1016/S1470-2045(18)30864-7 30518502 PMC6733402

[121] Shiqi L Jiasi Z Lvzhe C Huailong X Liping H Lin L . Durable remission related to CAR-T persistence in R/R B-ALL and long-term persistence potential of prime CAR-T. Mol Ther Oncolytics. (2023) 29:107–17. doi: 10.1016/j.omto.2023.04.003 37215385 PMC10196916

[122] University of Pennsylvania . Phase I Clinical Trial of Autologous Mesothelin Re-Directed T Cells in Patients With Chemotherapy Refractory Metastatic Pancreatic Cancer (2017). clinicaltrials.gov. Available online at: https://clinicaltrials.gov/study/NCT01897415 (Accessed May 24, 2026). Clinical trial registration NCT01897415.

[123] Hou X Zaks T Langer R Dong Y . Lipid nanoparticles for mRNA delivery. Nat Rev Mater. (2021) 6:1078–94. doi: 10.1038/s41578-021-00358-0 34394960 PMC8353930

[124] Cullis PR Felgner PL . The 60-year evolution of lipid nanoparticles for nucleic acid delivery. Nat Rev Drug Discov. (2024) 23:709–22. doi: 10.1038/s41573-024-00977-6 38965378

[125] Hassett KJ Benenato KE Jacquinet E Lee A Woods A Yuzhakov O . Optimization of lipid nanoparticles for intramuscular administration of mRNA vaccines. Mol Ther Nucleic Acids. (2019) 15:1–11. doi: 10.1016/j.omtn.2019.01.013 30785039 PMC6383180

[126] Sabnis S Kumarasinghe ES Salerno T Mihai C Ketova T Senn JJ . A novel amino lipid series for mRNA delivery: Improved endosomal escape and sustained pharmacology and safety in non-human primates. Mol Ther J Am Soc Gene Ther. (2018) 26:1509–19. doi: 10.1016/j.ymthe.2018.03.010 29653760 PMC5986714

[127] Ang MJY Metzloff AE Thatte AS Mitchell MJ . Lipid nanoparticles for engineering next generation CAR T cell immunotherapy. Nanoscale Horiz. (2026) 11:22–36. doi: 10.1039/d5nh00432b 41114533

[128] Drug products, including biological products, that contain nanomaterials: guidance for industry. Center for drug evaluation and research. Silver Spring, MD: U.S. Food and Drug Administration (2022).

[129] Hamilton AG Swingle KL Joseph RA Mai D Gong N Billingsley MM . Ionizable lipid nanoparticles with integrated immune checkpoint inhibition for mRNA CAR T cell engineering. Adv Healthc Mater. (2023) 12:2301515. doi: 10.1002/adhm.202301515 37602495

[130] Mukalel AJ Hamilton AG Billingsley MM Li J Thatte AS Han X . Oxidized mRNA lipid nanoparticles for in situ chimeric antigen receptor monocyte engineering. Adv Funct Mater. (2024) 34:2312038. doi: 10.1002/adfm.202312038 39628840 PMC11611297

[131] Shalek AK Robinson JT Karp ES Lee JS Ahn D-R Yoon M-H . Vertical silicon nanowires as a universal platform for delivering biomolecules into living cells. Proc Natl Acad Sci. (2010) 107:1870. doi: 10.1073/pnas.0909350107 20080678 PMC2836617

[132] Shalek AK Gaublomme JT Wang L Yosef N Chevrier N Andersen MS . Nanowire-mediated delivery enables functional interrogation of primary immune cells: Application to the analysis of chronic lymphocytic leukemia. Nano Lett. (2012) 12:6498–504. doi: 10.1021/nl3042917 23190424 PMC3573729

[133] Chiappini C Martinez JO De Rosa E Almeida CS Tasciotti E Stevens MM . Biodegradable nanoneedles for localized delivery of nanoparticles *in vivo*: Exploring the biointerface. ACS Nano. (2015) 9:5500–9. doi: 10.1021/acsnano.5b01490 25858596 PMC4733661

[134] Chiappini C Chen Y Aslanoglou S Mariano A Mollo V Mu H . Tutorial: Using nanoneedles for intracellular delivery. London: Nature Portfolio. doi: 10.1038/s41596-021-00600-7. 34426708

[135] Elnathan R Tay A Voelcker NH Chiappini C . The start-ups taking nanoneedles into the clinic. Nat Nanotechnol. (2022) 17:807–11. doi: 10.1038/S41565-022-01158-5 35760894

[136] Ekstrand F Mapar M Ruhrmann S Bacos K Ling C Prinz CN . Achieving efficient clonal beta cells transfection using nanostraw/nanopore-assisted electroporation. RSC Adv. (2024) 14:22244–52. doi: 10.1039/d4ra02791d 39010923 PMC11247384

[137] Hebisch E Hjort M Volpati D Prinz CN . Nanostraw-assisted cellular injection of fluorescent nanodiamonds via direct membrane opening. Small. (2021) 17:2006421. doi: 10.1002/smll.202006421 33502091

[138] Cao Y Chen H Qiu R Hanna M Ma E Hjort M . Universal intracellular biomolecule delivery with precise dosage control. Sci Adv. (2018) 4:eaat8131. doi: 10.1126/sciadv.aat8131 30402539 PMC6209385

[139] Eyquem J Mansilla-Soto J Giavridis T van der Stegen SJC Hamieh M Cunanan KM . Targeting a CAR to the TRAC locus with CRISPR/Cas9 enhances tumour rejection. Nature. (2017) 543:113–7. doi: 10.1038/nature21405 28225754 PMC5558614

[140] Wilbie D Walther J Mastrobattista E . Delivery aspects of CRISPR/Cas for *in vivo* genome editing. Acc Chem Res. (2019) 52:1555–64. doi: 10.1021/acs.accounts.9b00106 31099553 PMC6584901

[141] Glass Z Lee M Li Y Xu Q . Engineering the delivery system for CRISPR-based genome editing. Trends Biotechnol. (2018) 36:173–85. doi: 10.1016/j.tibtech.2017.11.006 29305085 PMC5801045

[142] Gattinoni L Klebanoff CA Restifo NP . Paths to stemness: Building the ultimate antitumour T cell. Nat Rev Cancer. (2012) 12:671–84. doi: 10.1038/nrc3322 22996603 PMC6352980

[143] Biasco L Scala S Basso Ricci L Dionisio F Baricordi C Calabria A . *In vivo* tracking of T cells in humans unveils decade-long survival and activity of genetically modified T memory stem cells. Sci Transl Med. (2015) 7:273ra13. doi: 10.1126/scitranslmed.3010314 25653219

[144] Lv J Si T Wang D Zhang C Zhou Y Yu J . Mechanical signaling via B2 integrin decouples T cell proliferation and differentiation for generating stem cell-like CAR T cells. Immunity. (2025) 58:2289–2304.e10. doi: 10.1016/j.immuni.2025.07.018 40782798

[145] Smith TT Stephan SB Moffett HF McKnight LE Ji W Reiman D . In situ programming of leukaemia-specific T cells using synthetic DNA nanocarriers. Nat Nanotechnol. (2017) 12:813–20. doi: 10.1038/nnano.2017.57 28416815 PMC5646367

[146] Jain M Est-Witte SE Shannon SR Neshat SY Yu X Dunham S . Biodegradable targeted polymeric mRNA nanoparticles enable *in vivo* CD19 CAR T cell generation and lead to B cell depletion. Sci Adv. (2026) 12:eadz1722. doi: 10.1126/sciadv.adz1722 41811961 PMC12978253

[147] Billingsley MM Gong N Mukalel AJ Thatte AS El-Mayta R Patel SK . *In vivo* mRNA CAR T cell engineering via targeted ionizable lipid nanoparticles with extrahepatic tropism. Small. (2024) 20:2304378. doi: 10.1002/smll.202304378 38072809

[148] Xu J Liu L Parone P Xie W Sun C Chen Z . In-vivo B-cell maturation antigen CAR T-cell therapy for relapsed or refractory multiple myeloma. Lancet. (2025) 406:228–31. doi: 10.1016/S0140-6736(25)01030-X 40617243

[149] An N Wang D Zhang P Zhang J Parone P Hu J . *In vivo* generation of anti-BCMA CAR-T cells in relapsed or refractory multiple myeloma: A phase 1 study. Nat Med. (2026) 32:1257–66. doi: 10.1038/s41591-026-04244-6 41882404

[150] Buggert M Vella LA Nguyen S Wu VH Chen Z Sekine T . The identity of human tissue-emigrant CD8+ T cells. Cell. (2020) 183:1946–1961.e15. doi: 10.1016/j.cell.2020.11.019 33306960 PMC9341432

[151] Albert BA Yang Y Shao XM Singh D Smit KN Anagnostou V . Deep neural networks predict class I major histocompatibility complex epitope presentation and transfer learn neoepitope immunogenicity. Nat Mach Intell. (2023) 5:861–72. doi: 10.1038/s42256-023-00694-6 37829001 PMC10569228

[152] Liu Y Cui C Liu L Cui Q . Enhancing mRNA translation efficiency with discriminative and generative artificial intelligence by optimizing 5′ UTR sequences. iScience. (2025) 28:113544. doi: 10.1016/j.isci.2025.113544 41069846 PMC12506572

[153] Li B Raji IO Gordon AGR Sun L Raimondo TM Oladimeji FA . Accelerating ionizable lipid discovery for mRNA delivery using machine learning and combinatorial chemistry. Nat Mater. (2024) 23:1002–8. doi: 10.1038/s41563-024-01867-3 38740955

[154] Cho Y Choi Y Jang Y Seong H . Nanomaterial‐enhanced biosensing: Mechanisms and emerging applications. Adv Healthc Mater. (2025) 14:2500189. doi: 10.1002/adhm.202500189 40167299 PMC12683224

[155] Mujawar MA Gohel H Bhardwaj SK Srinivasan S Hickman N Kaushik A . Nano-enabled biosensing systems for intelligent healthcare: Towards COVID-19 management. Mater Today Chem. (2020) 17:100306. doi: 10.1016/j.mtchem.2020.100306 32835155 PMC7274574

[156] Noah NM Ndangili PM . Current trends of nanobiosensors for point-of-care diagnostics. J Anal Methods Chem. (2019) 2019:2179718. doi: 10.1155/2019/2179718 31886019 PMC6925704

[157] Wang J . Electrochemical biosensors: Towards point-of-care cancer diagnostics. Biosens Bioelectron. (2006) 21:1887–92. doi: 10.1016/j.bios.2005.10.027 16330202

[158] Dincer C Bruch R Costa-Rama E Fernández-Abedul MT Merkoçi A Manz A . Disposable sensors in diagnostics, food, and environmental monitoring. Adv Mater. (2019) 31:1806739. doi: 10.1002/adma.201806739 31094032

[159] Mayer KM Hafner JH . Localized surface plasmon resonance sensors. Chem Rev. (2011) 111:3828–57. doi: 10.1021/cr100313v 21648956

[160] Anker JN Hall WP Lyandres O Shah NC Zhao J Van Duyne RP . Biosensing with plasmonic nanosensors. Nat Mater. (2008) 7:442–53. doi: 10.1038/nmat2162 18497851

[161] Jackman JA Ferhan AR Cho N-J . Nanoplasmonic sensors for biointerfacial science. Chem Soc Rev. (2017) 46:3615–60. doi: 10.1039/C6CS00494F 28383083

[162] Patolsky F Zheng G Lieber CM . Nanowire-based biosensors. Anal Chem. (2006) 78:4260–9. doi: 10.1021/ac069419j 16856252

[163] Cui Y Wei Q Park H Lieber CM . Nanowire nanosensors for highly sensitive and selective detection of biological and chemical species. Science. (2001) 293:1289–92. doi: 10.1126/science.1062711 11509722

[164] Gao N Zhou W Jiang X Hong G Fu T-M Lieber CM . General strategy for biodetection in high ionic strength solutions using transistor-based nanoelectronic sensors. Nano Lett. (2015) 15:2143–8. doi: 10.1021/acs.nanolett.5b00133 25664395 PMC4594804

[165] Giaever I Keese CR . Micromotion of mammalian cells measured electrically. Proc Natl Acad Sci USA. (1991) 88:7896–900. doi: 10.1073/pnas.88.17.7896 1881923 PMC52411

[166] Xing JZ Zhu L Gabos S Xie L . Microelectronic cell sensor assay for detection of cytotoxicity and prediction of acute toxicity. Toxicol Vitro. (2006) 20:995–1004. doi: 10.1016/j.tiv.2005.12.008 16481145

[167] Logun M Colonna MB Mueller KP Ventarapragada D Rodier R Tondepu C . Label-free *in vitro* assays predict the potency of anti-disialoganglioside chimeric antigen receptor T-cell products. Cytotherapy. (2023) 25:670–82. doi: 10.1016/j.jcyt.2023.01.008 36849306 PMC10159906

[168] Lamarche B Zhang J Yang G Ye P Li N Abassi Y . A multiparametric CAR T cell potency assay that employs real-time cellular impedance and live cell imaging. Cytotherapy. (2020) 22:S149. doi: 10.1016/j.jcyt.2020.03.30442574925

[169] Paterson K Zanivan S Glasspool R Coffelt SB Zagnoni M . Microfluidic technologies for immunotherapy studies on solid tumours. Lab Chip. (2021) 21:2306–29. doi: 10.1039/d0lc01305f 34085677 PMC8204114

[170] Sackmann EK Fulton AL Beebe DJ . The present and future role of microfluidics in biomedical research. Nature. (2014) 507:181–9. doi: 10.1038/nature13118 24622198

[171] Xiong Y Libby KA Su X . The physical landscape of CAR-T synapse. Biophys J. (2024) 123:2199–210. doi: 10.1016/j.bpj.2023.09.004 37715447 PMC11331049

[172] Zhang DKY Brockman JM Adu-Berchie K Liu Y Binenbaum Y de Lázaro I . Subcutaneous biodegradable scaffolds for restimulating the antitumour activity of pre-administered CAR-T cells. Nat BioMed Eng. (2025) 9:268–78. doi: 10.1038/s41551-024-01216-4 38831041

[173] Ma C Wang H Liu L Chen R Mukherjee N Tong J . Bioengineered immunocompetent preclinical trial-on-chip tool enables screening of CAR T cell therapy for leukaemia. Nat BioMed Eng. (2025) 9:2098–114. doi: 10.1038/s41551-025-01428-2 40595437 PMC12705464

[174] Abdo L Batista-Silva LR Bonamino MH . Cost-effective strategies for CAR-T cell therapy manufacturing. Mol Ther Oncol. (2025) 33:200980. doi: 10.1016/j.omton.2025.200980 40291594 PMC12022644

